# Disease activity and treatment response in early rheumatoid arthritis: an exploratory metabolomic profiling in the NORD-STAR cohort

**DOI:** 10.1186/s13075-025-03616-6

**Published:** 2025-07-26

**Authors:** Tahzeeb Fatima, Yuan Zhang, Georgios K. Vasileiadis, Araz Rawshani, Ronald van Vollenhoven, Jon Lampa, Bjorn Gudbjornsson, Espen A. Haavardsholm, Dan Nordström, Gerdur Gröndal, Kim Hørslev-Petersen, Kristina Lend, Marte S. Heiberg, Merete Lund Hetland, Michael Nurmohamed, Mikkel Østergaard, Till Uhlig, Tuulikki Sokka-Isler, Anna Rudin, Cristina Maglio

**Affiliations:** 1 Department of Rheumatology and Inflammation Research, Institute of Medicine, Sahlgrenska Academy, University of Gothenburg, Gothenburg, Sweden; 2 Department of Molecular and Clinical Medicine, Institute of Medicine, Sahlgrenska Academy, University of Gothenburg, Gothenburg, Sweden; 3 Wallenberg Laboratory for Cardiovascular and Metabolic Research, Institute of Medicine, University of Gothenburg, Gothenburg, Sweden; 4 Department of Medicine, Rheumatology Unit, Center for Molecular Medicine (CMM), Karolinska Institute, Karolinska University Hospital, Stockholm, Sweden; 5 Amsterdam Rheumatology and Immunology Center, Amsterdam University Medical Centers, Amsterdam, Netherlands; 6 Centre for Rheumatology Research, Landspitali University Hospital, Reykjavik, Iceland; 7 Faculty of Medicine, University of Iceland, Reykjavik, Iceland; 8 Center for treatment of Rheumatic and Musculoskeletal Diseases (REMEDY), Diakonhjemmet Hospital, Oslo, Norway; 9 Faculty of Medicine, University of Oslo, Oslo, Norway; 10 Division of Medicine and Rheumatology, Helsinki University Hospital, Helsinki, Finland; 11 University of Helsinki, Helsinki, Finland; 12 Danish Hospital for Rheumatic Diseases, University Hospital of Southern Denmark, Sønderborg, Denmark; 13 Department of Regional Health Research, University of Southern Denmark, Odense, Denmark; 14 Copenhagen Center for Arthritis Research (COPECARE) and DANBIO, Center for Rheumatology and Spine Diseases, Rigshospitalet, Glostrup, Denmark; 15 Department of Clinical Medicine, Faculty of Health Sciences, University of Copenhagen, Copenhagen, Denmark; 16 Amsterdam Rheumatology and immunology Center, Reade, Amsterdam, Netherlands; 17 Department of Medicine, University of Eastern Finland, Jyväskylä Central Hospital, Jyväskylä, Finland; 18 Rheumatology Clinic, Sahlgrenska University Hospital, Gothenburg, Sweden; 19 Wallenberg Centre for Molecular and Translational Medicine, University of Gothenburg, Gothenburg, Sweden

**Keywords:** Rheumatoid arthritis, Disease activity, Treatment response, Metabolomics, Machine learning

## Abstract

**Background:**

The variability in treatment response in people with rheumatoid arthritis (RA) warrants the prediction of patients at high risk of treatment failure. Identification of biomarkers linked to clinical remission in RA is currently a challenge. Metabolomics may help to identify such biomarkers as it allows for a comprehensive exploration of disease-related variations that extends beyond the genome and proteome. This hypothesis-free exploratory metabolomics study aimed to profile serum metabolic alterations in early RA to understand the metabolic changes associated with disease activity and therapeutic response.

**Methods:**

The study included 220 early RA participants from the NORD-STAR study, randomized at baseline into four arms, ranging from conventional anti-rheumatic treatment to biological drugs: methotrexate combined with prednisolone (1), certolizumab (2), abatacept (3), or tocilizumab (4). Untargeted metabolomics was performed in serum samples at baseline and 24-week follow-up. Participants achieving clinical disease activity index remission at 24 weeks were defined as responders. Machine learning models for treatment response were constructed using random forest, logistic regression, support vector machine and extreme gradient boosting algorithms based on selected features.

**Results:**

We identified 278 metabolites, of which 39 were associated with baseline disease activity, including several acylcarnitines and amino acids. We also found 17 baseline metabolites associated with remission at 24 weeks in the overall cohort, including malic acid (β=-0.4), cytidine (β = 0.4), arginine (β = 0.3), and citrulline (β = 0.2), as well as specific metabolites and metabolic pathways associated with remission in the four treatment arms. Fifteen features were identified using machine learning-based multivariable selection. The best predictive model using logistic regression achieved AUC of 0.75 in training and 0.73 in the test set.

**Conclusions:**

Our study has identified several baseline metabolites and metabolic pathways associated with disease activity and response to different treatments in early RA. By integrating metabolomics and clinical data, we developed predictive models for response to treatment in early RA, though their predictive performance remains limited.

**Supplementary Information:**

The online version contains supplementary material available at 10.1186/s13075-025-03616-6.

## Background

Rheumatoid arthritis (RA) is a common rheumatic disease with a worldwide estimated prevalence of 0.46%, and is characterized by progressive joint damage and the presence of extra-articular manifestations [1, 2]. Despite several treatment alternatives, there is no permanent cure [3]. Patients’ response to treatment exhibits significant variability and around 50% of people with early RA do not achieve remission at 6-month follow-up [4]. Treatment failure is not only associated with the risk of developing enduring complications but is also linked with a reduced quality of life and places a financial burden on the society [5, 6], which would advocate for the identification of predictors of treatment response in RA.

Metabolomics is the study of the intermediate and end products of metabolism. Among the -omics disciplines, metabolomics best reflects phenotype, as the metabolome is highly dynamic and rapidly responds to environmental factors [7, 8]. Several recent studies have employed combination of mass spectrometry (MS)-based metabolomics with machine learning algorithms to identify potential metabolite biomarkers for disease diagnosis and clinical presentations [9, 10]. Only a small number of studies have previously explored changes in circulating metabolites in RA, primarily in the context of diagnosis with even fewer focusing on treatment response [11–16]. These studies had either a limited list of metabolites identified or were mostly performed in individuals with established RA, potentially introducing the influence of long-term disease duration and prior treatments on the outcomes. Data on the metabolomic profiles of people with newly diagnosed RA is scarce. A previous study has identified a distinct metabolic fingerprint of 16 treatment-naïve people with RA in comparison to healthy controls [17], whereas a targeted analysis focusing on specific metabolic pathways performed in 60 participants with early RA has described circulating metabolites associated with sustained drug-free remission [18]. Furthermore, a larger metabolomics analysis in serum and urine samples has identified metabolites associated with c-reactive protein (CRP) in treatment-naïve people with RA [19]. To date, the serum metabolome has never been studied in a large cohort of people with newly diagnosed RA to identify metabolites associated with disease activity and response to treatment.

In this study, we aimed to address this gap by studying the serum metabolomics profile of 220 participants with untreated early RA from the Nordic Rheumatic Diseases Strategy Trials and Registries (NORD-STAR) study [4, 20]. Using high-performance MS-based untargeted metabolomics, we sought to identify metabolites associated with disease activity and treatment response to uncover molecular patterns in early RA. We further developed prediction models of treatment response in people with untreated early RA by using four machine learning algorithms based on selected metabolites and clinical features. Rather than claiming direct clinical applicability, this exploratory study aimed to contribute to a deeper understanding of RA by giving insights on the impact of metabolism on disease activity and response to treatment.

## Methods

### Study design and participants

NORD-STAR is a multicenter, randomized, open-label, blinded-assessor, phase 4 longitudinal trial, designed to compare efficacy and safety of active conventional anti-rheumatic treatment vs. three different biological disease modifying anti-rheumatic drugs in people with untreated early RA. The complete details of the selection criteria of the participants in the NORD-STAR study are provided elsewhere [4].

For this report, we acquired the data of 393 participants that constituted the Swedish arm of the NORD-STAR study cohort. Data were collected at baseline and after 24 weeks of treatment. At baseline, study participants were randomized (1:1:1:1) to four treatments that all included methotrexate up to 25 mg/week. Participants in arm 1 (or active conventional treatment) also received oral prednisolone (tapered from 20 to 5 mg/day in nine weeks). Participants in arm 2 received certolizumab-pegol at a subcutaneous dose of 200 mg every other week (400 mg at week 0, 2, and 4). In arm 3, participants received abatacept at 125 mg every week subcutaneously, while those in arm 4 received tocilizumab at 8 mg/kg every four weeks intravenously or 162 mg each week subcutaneously. After the assessment for the availability of serum, completeness of data for several study variables, and a random stratification to have reasonable numbers in each treatment arm, a subset of 220 participants was selected to perform the metabolomic profiling. The distribution of participants was 58, 52, 56, and 54 for arms 1, 2, 3, and 4, respectively. The primary end-point of this report was response to treatment at 24 weeks defined by Clinical Disease Activity Index remission (CDAI ≤ 2.8), which is also the primary outcome for the NORD-STAR study [4]. Participants achieving CDAI remission at 24-week follow-up were defined as “responders”, whereas those who did not achieve remission were defined as “non-responders”.

### MS metabolomics analysis

To run the metabolomics analysis in 220 selected participants, serum samples were obtained from the NORD-STAR study sample collection [4]. The samples were then aliquoted (100 µl per sample) and stored at -80 °C before transportation in dry ice to the Swedish Metabolomics Centre in Umeå, Sweden, where untargeted metabolomics was performed using ultra-high performance liquid chromatography-quadrupole time-of-flight mass spectrometry (UHPLC-QTOF-MS). Following the attainment of peak intensities for the untargeted data, a targeted pre-processing approach was employed to identify the list of metabolites that were pre-annotated. The current report utilized the peak intensities data of only pre-annotated metabolites for further analysis (Supplementary text).

### Statistical analysis

**Fig. 1:**
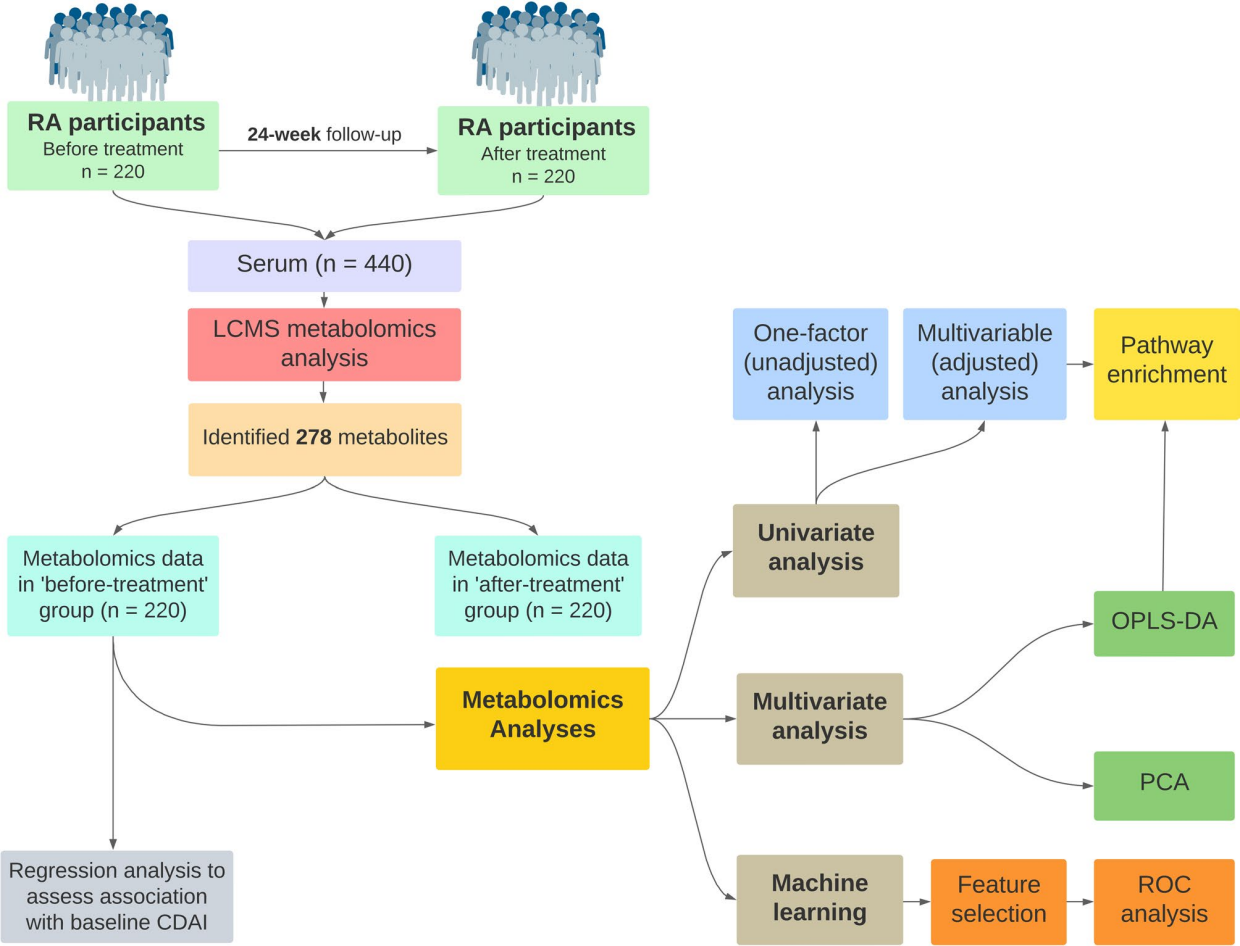
Study design and data analysis workflow. RA: early rheumatoid arthritis, LC-MS: Liquid chromatography-mass spectrometry, CDAI: Clinical Disease Activity Index, PCA: principal component analysis, OPLS-DA: orthogonal partial least square discriminant analysis, ROC: receiver operating characteristic

Figure 1 illustrates the study design and data analysis workflow for this study. The raw peak intensities data for pre-annotated metabolites were normalized against the internal standards to address the difference between different run batches on LC-MS. Autoscaling, which involves centering around the mean and dividing by the standard deviation of each variable, was performed before any further analyses [21]. All analyses were performed using MetaboAnalyst 6.0 [21] and R v4.3.2 [22]. Wherever applicable, an FDR (false discovery rate) correction (*p* < 0.05) was applied in the analyses to account for multiple testing. Associations with raw *p* < 0.05 but above the FDR-corrected threshold were deemed suggestive, whereas those passing the FDR correction were considered significant.

We first fitted a fixed effects multivariable linear regression model to assess the relationship between metabolites and disease activity at baseline. The regression model was further adjusted for age, sex, and body mass index (BMI). To predict remission at 24 weeks, baseline peak intensity values of metabolites were utilized in all analyses [21]. To maximize the statistical power and to identify generalizable metabolic patterns, data analyses were primarily conducted in the entire dataset (*n* = 220). While this approach was adapted to emphasize the exploratory nature of the analysis, treatment stratified analyses also followed to explore more treatment specific metabolic patterns. An unsupervised principal component analysis (PCA) was performed to explore the global metabolic variations between responders and non-responders, followed by a supervised orthogonal partial least squares discriminant analysis (OPLS-DA) to maximize inter-group variations and to identify metabolites that significantly contributed to the observed variation [23]. A variable importance in projection (VIP) score, which is a measure of a variable’s importance in the OPLS-DA model, of ≥ 2 was considered to indicate the metabolites that influenced the group separation the most [24]. A power analysis was performed to calculate the minimum sample size required to obtain 95% power assuming probabilistic principal component analysis (PPCA) as model, with an FDR (false discovery rate) of 0.05, 300 bins and 0.2 as expected proportion of significant bins in an untargeted analysis using MetSizeR package in R [25].

We performed one-factor (unadjusted) and multivariable (covariate-adjusted) analyses as primary univariate analyses to, respectively, compare metabolites between responders and non-responders and to account for the variability associated with several covariates that could otherwise affect the association between metabolites and outcome of interest (CDAI remission at 24 weeks). The results from one-factor analysis (t-test and fold change or FC) were combined in a volcano plot using a *p* ≤ 0.05 and an FC of > 1.2 or < 0.83 as cut-offs. Multivariable analyses were performed with two sets of covariates using fixed effect generalized linear models or GLMs. Baseline covariates adjusted for in the primary multivariable analysis included age, sex, BMI, anti-citrullinated protein antibody (ACPA) status, treatment randomization, current smoking status and disease activity score of 28 joints based on CRP (DAS28-CRP). To ensure the robustness of the findings, a sensitivity multivariable analysis was conducted adjusting for baseline CDAI and CRP in place of DAS28-CRP, as CDAI better reflects physician global assessment, which is absent from DAS28. As paired serum samples (baseline and 24-week follow-up) were available, we calculated the relative change (or delta) from day 0 to week 24 for the metabolites that showed a significant association with CDAI remission after covariate adjustments to assess the longitudinal changes in the levels of these metabolites. These delta changes were stratified by treatment response status (responders vs. non-responders) to evaluate differential metabolic trajectories over time. The percentage delta was calculated as ‘[(value at 24 weeks - value at day 0) / value at day 0] x 100’. We also used paired t-test (*p* ≤ 0.05) to calculate the difference in levels of these metabolites between pretreatment (day 0 or baseline) and post treatment (at 24 weeks) groups.

For pathway enrichment analysis, all metabolites with a VIP score of ≥ 2 in OPLS-DA and *p* ≤ 0.05 in the primary multivariable analysis were utilized. These metabolites were mapped on The Small Molecule Pathway Database (SMPDB) to assess the significantly perturbed metabolic pathways. The pathway analysis used hypergeometric test for the over-representation analysis and node importance measure with relative betweenness centrality for the topological analysis.

To predict treatment response in early RA, we developed supervised machine learning models using baseline clinical and metabolomic data. Prior to any modeling, the dataset was randomly split into a training set (75%) and an independent test set (25%), which was held out entirely from feature selection and model training. All metabolites, along with a predefined set of clinical variables (age, sex, BMI, ACPA status, treatment randomization, current smoking status, and DAS28-CRP), were included as candidate predictors. Feature selection was performed using the MUVR2 (multivariate methods with Unbiased Variable selection in R version 2) algorithm [26], applied exclusively to the training data. MUVR2 employs repeated cross-validation within its framework to minimize overfitting and promote model generalizability. To assess feature stability, we conducted 1,000 bootstrap resampling iterations using the ‘rsample’ package. In each bootstrap, a random forest model within MUVR2 was trained to select informative variables. Feature selection frequencies were aggregated across all bootstraps, and the 15 most frequently selected variables were retained for downstream modeling. These features were then used to train four classification algorithms – logistic regression, support vector machine (SVM), random forest, and extreme gradient boosting (XGB) – again using only the training set. A 5-fold cross validation repeated five times was applied to compare a range of competing models. The predictive performance of each of these models was then evaluated on an exclusively held-out test dataset (25% of data) that had not been used at any stage of feature selection or model training. To access the added predictive value of the metabolites, we also built a baseline model using only selected clinical variables (age, sex, BMI, ACPA status, treatment randomization, current smoking status and DAS28-CRP), explicitly using same training and test datasets, and applying the fours machine learning algorithms as mentioned above. The area under the receiver operating characteristic curve (ROC-AUC) was used to assess the overall discriminatory performance of the model.

## Results

### Characteristics of the study participants

Table 1 summarizes the characteristics of the 220 participants included in the current report, who were randomly selected out of 393 participants from the Swedish cohort of the NORD-STAR trial. The anthropometric and clinical characteristics of these participants were similar to those who were not included in the present report (*n* = 173) (Additional file 1: Table S1). Of these 220 participants, 98 (44.5%) achieved CDAI remission at 24-week follow-up (responders) and 122 (55.4%) did not (non-responders) (Table 1). Responders had lower baseline BMI, number of tender joints, as well as lower markers of disease activity such as CDAI, DAS28 based on erythrocyte sedimentation rate (DAS28-ESR), and DAS28-CRP. Responders were more likely to be rheumatoid factor positive. Average levels of ESR and CRP were similar in both groups (Table 1).

**Table 1:** Baseline characteristics of people with early RA included in the current report, overall and after stratification according to response to treatment at the 24-week follow-up

Characteristics	All (*n* = 220)	Responders (*n* = 98)	Non-responders (*n* = 122)	*p*-value
Female, n (%)	153 (70)	61 (62)	92 (75)	0.06
Age, years	54 ± 15	52 ± 15	56 ± 14	0.05
BMI	26 ± 5	24 ± 3	26 ± 5	0.001
Current smokers, n (%)	44 (20)	18 (18)	26 (21)	0.64
RF positive, n (%)	162 (74)	78 (79)	84 (68)	0.04
ACPA positive, n (%)	180 (82)	85 (86)	95 (77)	0.05
Symptom duration, days	215 ± 169	191 ± 140	233 ± 187	0.07
Time since diagnosis, days	8 ± 26	8 ± 19	8 ± 30	0.99
ESR, mm/h	34 ± 25	34 ± 24	34 ± 24	0.86
CRP, mg/L	22 ± 31	22 ± 30	22 ± 31	0.91
SJC28	8 ± 5	8 ± 4	9 ± 5	0.09
TJC28	10 ± 6	8 ± 5	11 ± 7	< 0.001
DAS28-ESR	5.5 ± 1.1	5.3 ± 1.0	5.7 ± 1.2	0.002
DAS28-CRP	5.1 ± 1.1	4.8 ± 1.0	5.3 ± 1.1	0.001
CDAI	29 ± 12	25 ± 9	32 ± 13	< 0.001

Continuous variables are expressed as mean ± standard deviation, while categorical variables are expressed as number and percentages. Student t-test was used to compare continuous variables while Chi-square test was used to compare categorical variables between responders and non-responders. *p*-values are provided for a comparison between these two groups with a *p* ≤ 0.05 indicating significance

BMI: body mass index, RF: rheumatoid factor, ACPA: anti-citrullinated peptide antibody, ESR: Erythrocyte sedimentation rate, CRP: C-reactive protein, SJC28: Swollen joint count (out of 28), TJC28: Tender joint count (out of 28), DAS28-ESR: Disease activity score of 28 joints (ESR-based), DAS28-CRP: Disease activity score using 28 joint counts (CRP-based), CDAI: Clinical Disease Activity Index

### Serum metabolomic profiling in early RA

A total of 278 putatively annotated metabolites were identified in serum samples of participants with early RA at both baseline and 24 weeks after treatment (Fig. 1). An unsupervised PCA showed a clear separation of extraction blanks from other samples and a close clustering of quality control (QC) samples (Additional file 1: Figure S1A). This indicated that the metabolomics data was of good quality and had a high reproducibility. The targeted pre-processing approach identified a number of metabolite classes that, among others, included several amino compounds, benzenoids, bile acids, carbohydrates, acylcarnitines, fatty acids, keto acids, nucleotides, purines, pyridines, peptides, and steroids (Additional file 1: Figure S1B). The peak intensities data of the 278 identified metabolites included both metabolites in positive (*n* = 172) and negative (*n* = 106) ionization modes (Figure Additional file 1: S1C).

### Association of baseline metabolites with baseline CDAI

**Fig. 2:**
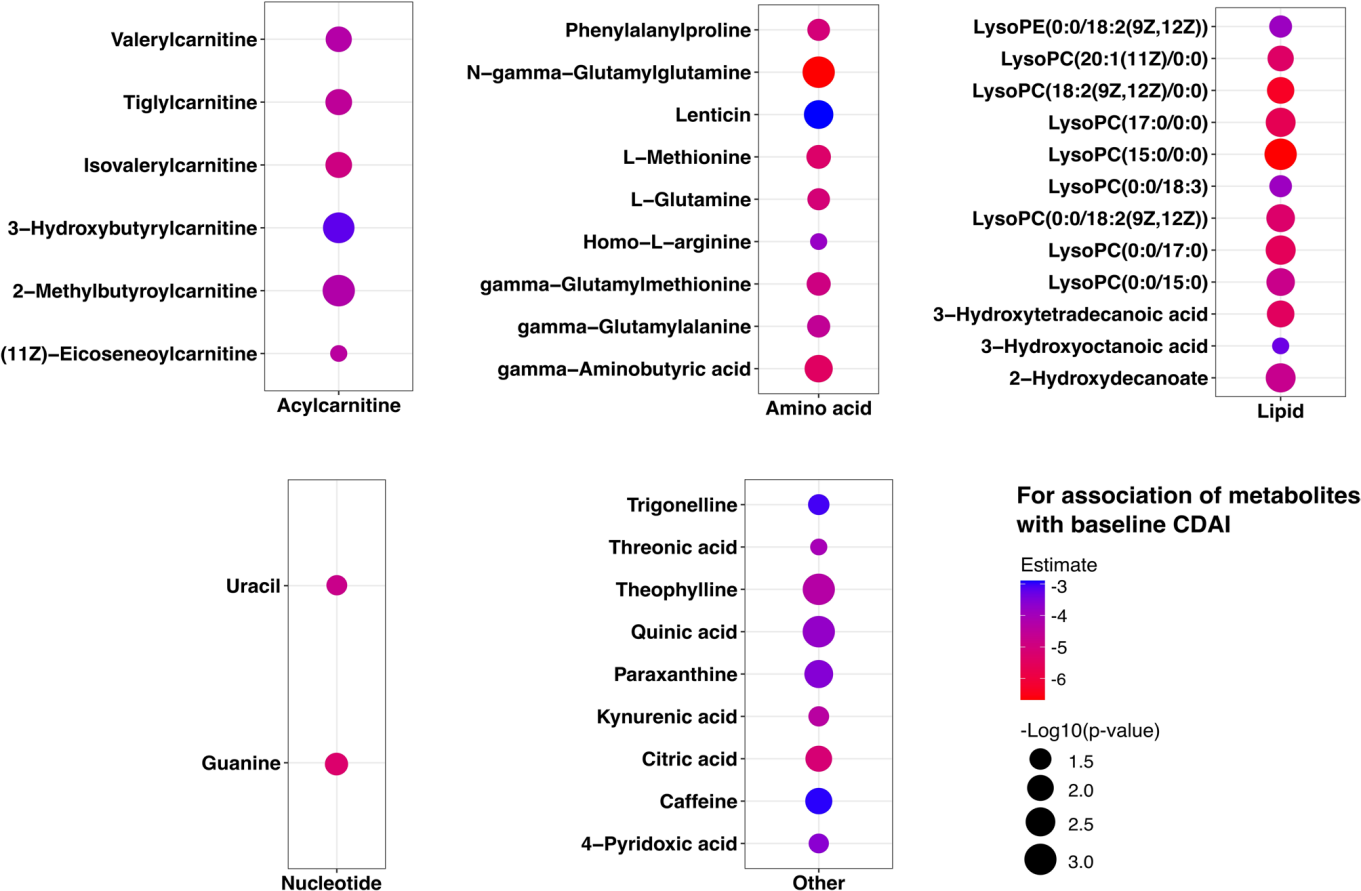
Dot plots illustrating the results from regression analysis (in 220 early RA participants) of the metabolomics data at baseline to assess their association with baseline CDAI (Clinical Disease Activity Index). The plots are stratified in the panel based on metabolite class categories, with each class indicated on the x-axis of each plot. RA: rheumatoid arthritis

In total, 39 metabolites were found to be significantly associated with baseline CDAI in multivariable linear regression model adjusted for age, sex, and BMI, with only acetaminophen (paracetamol) showing a positive association (β = 1.64, *p* = 0.03). Among the 38 metabolites inversely associated with baseline CDAI, six were acylcarnitines, and nine were amino acids, together with lipids, nucleotides, and other molecules (Fig. 2, Additional file 1: Table S2).

### Baseline serum metabolomic profiling according to response to treatment at the 24-week follow-up

**Fig. 3:**
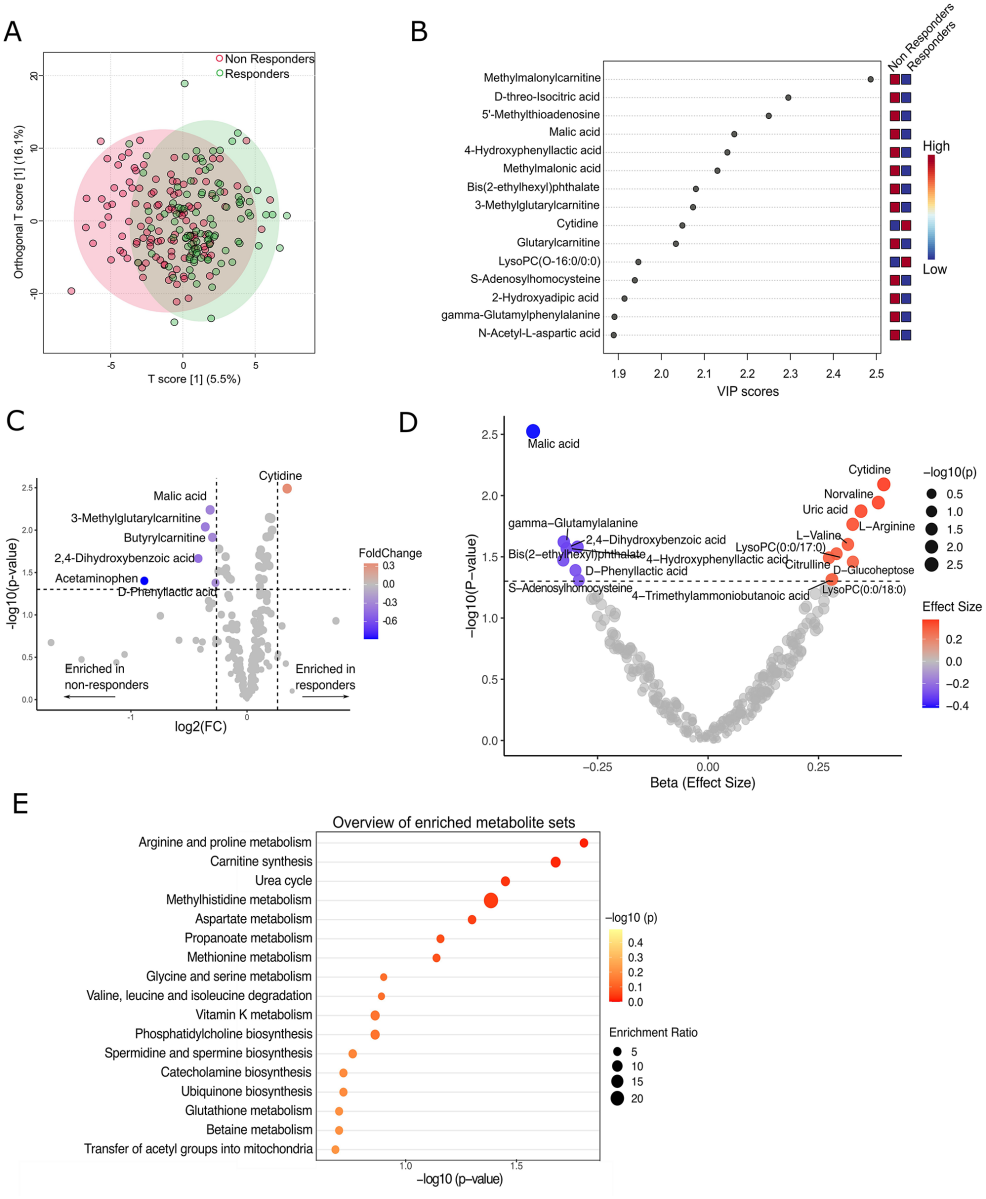
Metabolic profiles discriminating participants who responded to treatment at 24 weeks follow-up (responders, *n* = 98) from those who did not response to treatment at week 24 (non-responders, *n* = 122); (**A**) Score plot from orthogonal partial least squares discriminant analysis (OPLS-DA). Samples in the encircled areas are within the 95% confidence interval, (**B**) Variable importance in projection (VIP) score plots of metabolites derived from OPLS-DA illustrating the most important metabolites that discriminated responders from non-responders. Metabolite levels shown in the VIP plot represent relative abundances based on normalized intensity values, (**C**) Volcano plot of differential metabolites obtained from one-factor analyses. Metabolites with a fold change of > 1.2 and a t-test *p* ≤ 0.05 were considered significantly upregulated/enriched in responders, while metabolites with a fold change of < 0.83 and a t-test *p* ≤ 0.05 were considered significantly upregulated/enriched in non-responders. The *p*-values are not adjusted, (**D**) Results from primary multivariable (covariate adjusted) analysis for association of baseline metabolites with CDAI remission at 24 weeks, adjusted for age, sex, body mass index (BMI), anti-citrullinated protein antibody status, current smoking status, baseline Disease Activity Score with 28 joint counts-C-reactive protein based (DAS28-CRP), and treatment randomization, (**E**) Pathway enrichment plot in relation to response to treatment. D-Glucoheptose, LysoPC(0:0/17:0) and Bis(2-ethylhexyl)phthalate were not found in any database or did not have Human Metabolome Database (HMBD) id. CDAI: Clinical Disease Activity Index. Note: The metabolites identified in 3B (OPLS-DA) represent those that contribute most to group separation based on multivariate projection modelling, while those in 3 C (volcano plot) reflect statistically significant changes between groups based on univariate comparisons. Due to differences in statistical frameworks and variable selection criteria, not all metabolites identified in 3 C will necessarily appear in 3B, and vice versa

PCA analysis showing the metabolomic profile variation between responders and non-responders is illustrated in Additional file 1: Figure S2A. Principal components 1 to 5 explained 44.6% of the variance of the LC-MS data (Additional file 1: Figure S2B and C). The power analysis assuming PPCA as statistical model showed that a minimum sample size of 34 (*n* = 15 and 19 in two groups) was required to obtain 95% statistical power in the analysis. A supervised OPLS-DA revealed a better metabolite separation between responders and non-responders compared to PCA (Fig. 3A). Among the ten metabolites that achieved a VIP score of ≥ 2 in OPLS-DA, only cytidine was found to be higher in responders than non-responders, while all nine other metabolites were lower in responders than non-responders (Fig. 3B). One-factor analysis identified seven metabolites significantly different between the two groups (Fig. 3C). Of these, cytidine was found to be significantly up-regulated in responders, whereas malic acid, 3 methylglutarylcarnitine, butyrylcarnitine, 2,4-dihydroxybenzoic acid, acetaminophen, and D-phenyllactic acid were significantly down-regulated in responders compared to non-responders (Fig. 3C, Additional file 1: Table S3). The primary multivariable analysis showed a total of 17 baseline metabolites being significantly associated with CDAI remission at 24-weeks. These metabolites included malic acid (β = -0.4, *p* = 0.003), cytidine (β = 0.4, *p* = 0.008), norvaline (β = 0.4, *p* = 0.01), L-arginine (β = 0.3, *p* = 0.01), citrulline (β = 0.2, *p* = 0.03), and uric acid (β = 0.4, *p* = 0.01; Fig. 3D and Additional file 1: Table S4). The results for the sensitivity multivariable analysis were largely consistent with the primary analysis (Additional File 1: Figure S5). However, none of the above associations in both univariate (one-factor and multivariable) analyses passed the FDR correction threshold (Additional file 1: Table S3 and S4), indicating that while they may be biologically relevant, they should be interpreted with caution.

The pathway enrichment analysis indicated several metabolic pathways to be perturbed at baseline between responders and non-responders. These included, among others, arginine and proline metabolism, carnitine synthesis, malate-aspartate shuttle, urea cycle, transfer of acetyl groups into mitochondria, aspartate metabolism, and methionine metabolism (Fig. 3E).

**Fig. 4:**
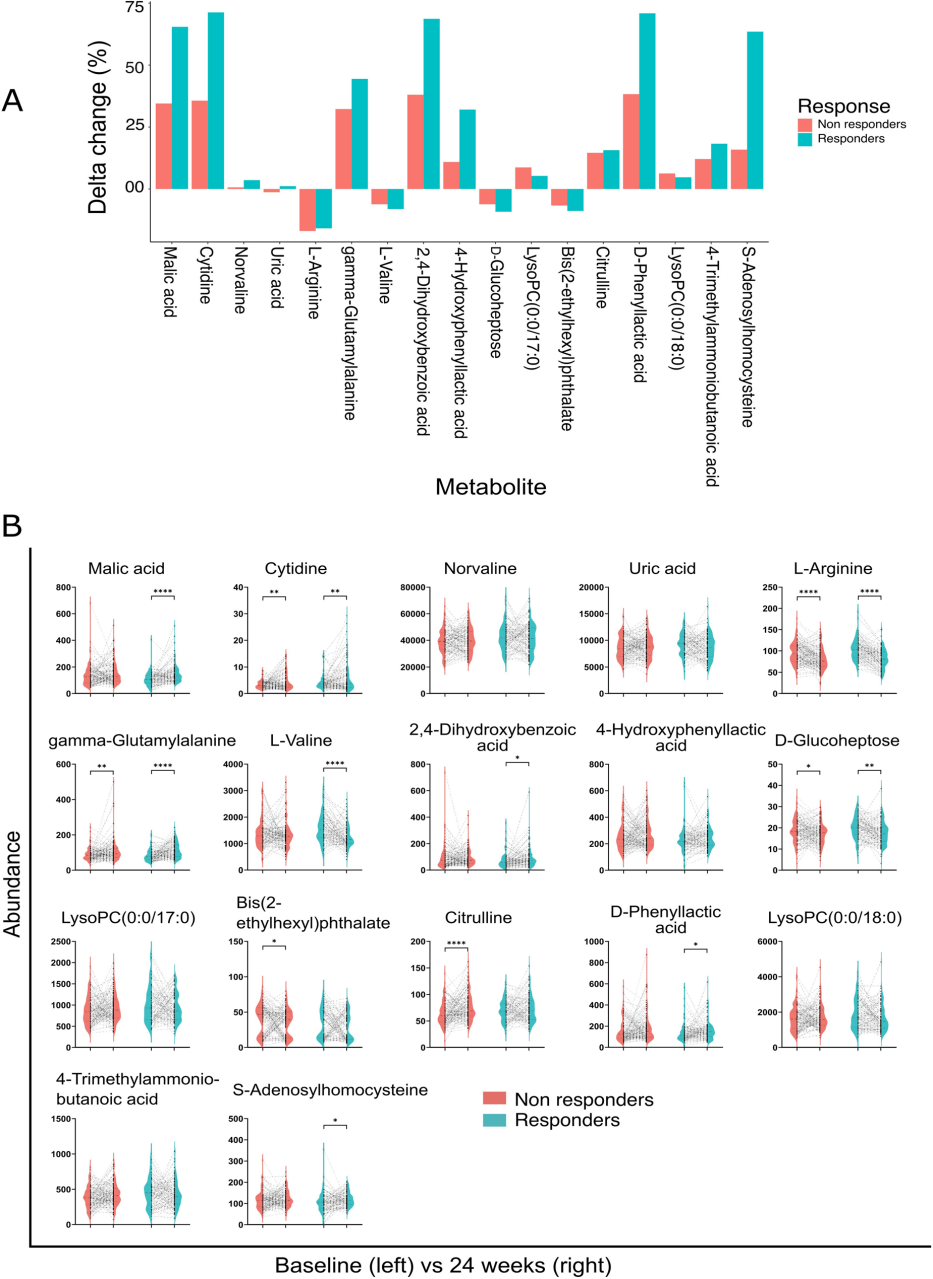
Results from (**A**) relative change (delta in percentage) in metabolites’ abundance between pre (baseline or day 0) and post (24 weeks) treatment groups stratified between responders and non-responders, and (**B**) Violin plots to illustrate results of paired t-test to indicate any change in metabolite abundance between pre- (baseline or at day 0) and post treatment (at 24 weeks) groups, stratified by responders and non-responders. * *p* < 0.05, ** *p* < 0.01, *** *p* < 0.001. Note: Only the significant metabolites predictive for CDAI remission at week 24 in primary multivariable analysis (*n* = 17) were plotted for this comparison. CDAI: Clinical Disease Activity Index

We also calculated a delta change, for both responders and non-responders, in the levels of 17 metabolites that showed significant association with treatment response in primary multivariable analysis (Fig. 4A). The results of paired t-test showed a significant change in several important metabolites from baseline to 24 weeks in both responders and non-responders, such as malic acid, cytidine, L-arginine, L-valine, and citrulline (Fig. 4B).

### Serum metabolomic profiling stratified by treatment arms

**Fig. 5:**
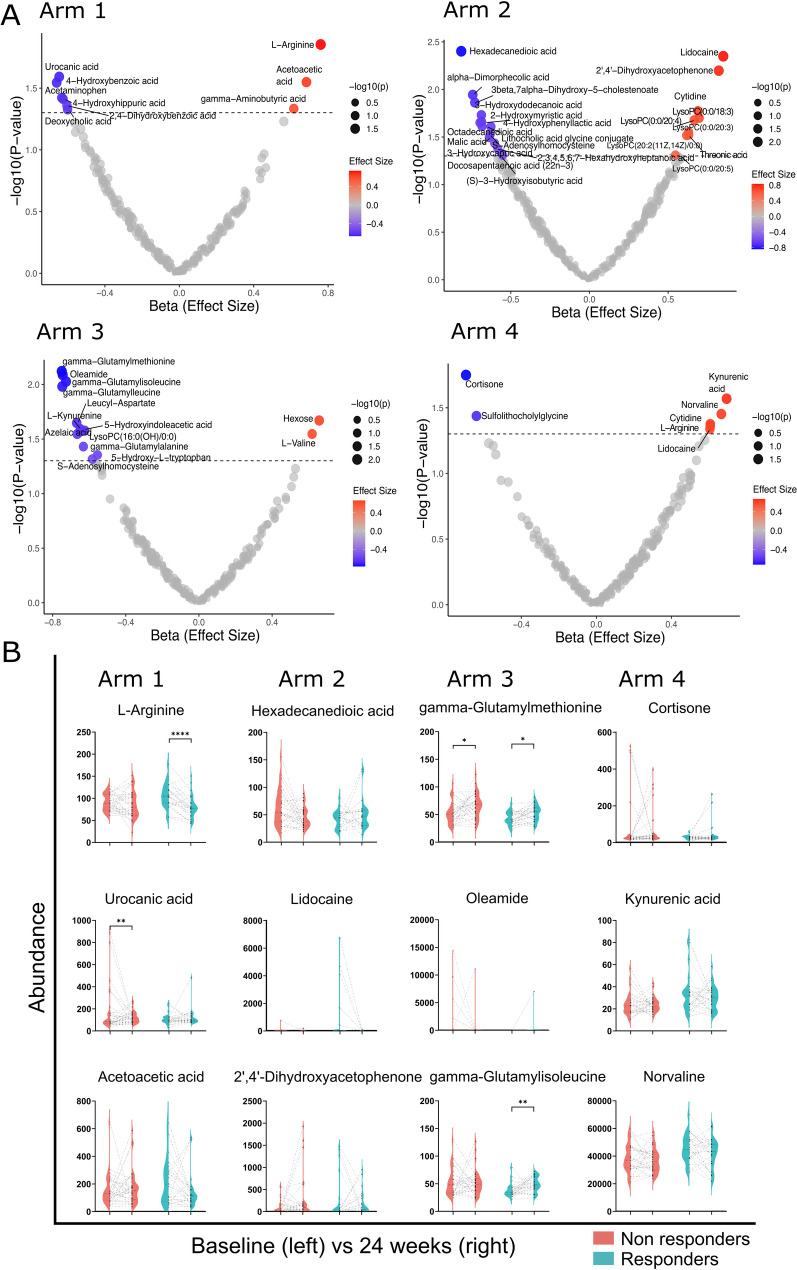
(**A**) Results from primary multivariable (covariate adjusted) analysis for association of baseline metabolites with CDAI remission at 24 weeks in four treatment arms (1 to 4). The model is adjusted for age, sex, anti-citrullinated protein antibody status, current smoking status, baseline Disease Activity Score with 28 joint counts-C-reactive protein based (DAS28-CRP), and treatment randomization. (**B**) Violin plots illustrating the results of paired t-test to indicate any change in normalized metabolite abundance between pre- (baseline or at day 0) and post treatment (at 24 weeks) groups. * *p* < 0.05, ** *p* < 0.01, *** *p* < 0.001. For each treatment arm, top three metabolites predictive for CDAI remission at week 24 in primary multivariable analysis were plotted for this comparison. CDAI: Clinical Disease Activity Index

We then stratified the cohort by the treatment arms to identify metabolites specifically associated with CDAI remission under different conditions. Results from PCA and OPLS-DA for each treatment arm are shown in Additional file 1: Figure S3. Overall, supervised OPLS-DA showed better separation between responders and non-responders in all treatment arms. Additional file 1: Figure S4 shows the OPLS-DA VIP score plots for the metabolites and the volcano plots from the one-factor analyses for each treatment arm. The primary multivariable (adjusted) analysis identified several baseline metabolites associated with CDAI remission at 24 weeks (Fig. 5A). For arm 1 (active conventional treatment arm), nine baseline metabolites were significantly associated with CDAI remission at 24 weeks, including L-arginine, acetaminophen, and urocanic acid. For arm 2 (certolizumab-pegol), the multivariable analysis identified 23 metabolites significantly associated with 24-week CDAI remission, including hexadecanedioic acid, cytidine, and malic acid. Fourteen baseline metabolites have been associated with 24-week CDAI remission after adjustment in arm 3 (abatacept), including oleamide, gamma-glutamylmethionine, L-valine and leucyl-aspartate. For arm 4 (tocilizumab), seven baseline metabolites were associated with 24-week CDAI remission after adjustment, including kynurenic acid, norvaline, cytidine and L-arginine (Fig. 5A, Tables S5-8). For each treatment arm, top three metabolites showing significant association in the primary multivariable analysis were evaluated for a difference between pre (day 0) and post treatment (24 weeks) groups using paired t-test (*p* ≤ 0.05, Fig. 5B). Results for pathway enrichment and topology analysis are shown in Additional file 1: Figure S6. However, none of these associations remained significant after FDR correction (Additional file 1: Tables S5-8), suggesting that these findings are exploratory and require further validation.

### Machine learning modelling to predict a response to treatment in early RA

Bootstrapping across 1,000 iterations in the training dataset revealed notable variability in feature selection. From this, the 15 most consistently selected features were identified and retained for downstream predictive modeling (Additional file 1: Figure S7A). Re-modelling these features using logistic regression achieved an AUC of 0.75 in the training set and 0.73 in the test set, as determined by ROC analysis (Additional file 1: Figure S7B). For the four different machine learning algorithms the AUC-ROC values ranged from 0.75 to 0.79 in cross-validated training dataset, and from 0.55 to 0.73 when evaluated in completely held-out test dataset for the final models (Table 2,). In comparison, models trained using only the selected clinical variables (age, sex, BMI, ACPA status, treatment allocation, current smoking status, and DAS28-CRP) achieved lower performance, with AUC-ROC ranging from 0.65 to 0.69 in training and 0.54 to 0.61 in test data.

**Table 2:** Results from different machine learning algorithms used to model selected features

Model	ROC-AUC in each modelling algorithm							
	Logistic regression		SVM		Random forest		XG-boost	
	Train	Test	Train	Test	Train	Test	Train	Test
Clinical variables only	0.69	0.54	0.69	0.57	0.65	0.57	0.67	0.61
Clinical & metabolites	0.75	0.73	0.76	0.72	0.79	0.56	0.75	0.55

For clinical variables only, the best random forest model encompassed 500 trees with mtry 1 and min-n 10, while the best XGB encompassed 500 trees, with maximum tree depth 11, mtry 1, min-n 6, learn rate 4.2e-3, loss reduction 0 and sample size 1. For clinical & metabolites, the best random forest model encompassed 947 trees with mtry 2 and min-n 6, while the best XGB model encompassed 1057 trees, with maximum tree depth 2, mtry 15, min-n 3, learn rate 0.002, loss reduction 0, and sample size 0.8. ROC-AUC: area under the receiver operating characteristic curve, SVM: support vector machine XG-boost: extreme gradient boosting

## Discussion

This hypothesis-free exploratory report presents a detailed serum metabolomic profiling of patients with untreated early RA and its association with disease activity and treatment response. At baseline, circulating levels of several acylcarnitines and amino acids were found to be inversely associated with disease activity. Moreover, circulating levels of malic acid, L-arginine, citrulline, and cytidine and their related pathways were associated with treatment response in early RA. We have also integrated metabolomics and clinical data to create predictive models for response to treatment that outperformed the clinical-only one, although both showed limited predictive value.

In all analyses, pre-annotated metabolomics data have been used, to ensure heightened structural and functional validity of the metabolites. First, we performed exploratory regression analyses of the baseline metabolomics data to detect associations between metabolites and baseline CDAI in adjusted model. Acetaminophen, or paracetamol, levels in serum were positively associated with baseline disease activity. Acetaminophen, a painkiller, is commonly used for pain management, and its serum levels indicate higher consumption in patients with elevated disease activity. This correlation positions acetaminophen levels as a reliable positive control in our analysis. Conversely, the other 38 metabolites were inversely associated with disease activity. Among these, six were acylcarnitines, i.e., fatty acid metabolites created by the conjugation of a fatty acid with L-carnitine [27]. The main function of acylcarnitines is to move acyl groups from the cytosol into the mitochondria to start β-oxidation in order to produce energy [27]. Previous studies in participants with established RA have reported elevated levels of acylcarnitine in lower disease activity [28, 29] and an increase in serum levels of acylcarnitine in established RA compared to healthy controls [14]. A more recent study in a women-based cohort reported an inverse association between plasma levels of three short-chain acylcarnitines and incidence of RA [30]. Data regarding the effect of treatment on acylcarnitine levels is lacking, with only one study reporting a reduction in levels of acylcarnitine following methotrexate therapy in RA [14]. Our findings, coupled with previous studies highlighting alterations in acylcarnitine levels, indicate a notable disturbance in fatty acid metabolism during active disease in people with RA. This is suggestive for a deviation from the typical glucose metabolism known to be associated with RA [31].

The other metabolites that we have found inversely associated with disease activity at baseline primarily included amino acids, lipids, and nucleotides and they were part of urea cycle, amino acid metabolism, caffeine metabolism, arginine and proline metabolism, citric acid cycle as well as pyrimidine and purine metabolisms. Among them, citric acid and methionine have also been previously identified as associated with disease activity in a cohort of 49 people with established RA who were naïve to biological anti-rheumatic drugs [16].

We then determined which serum metabolites were altered at baseline in those who achieved CDAI remission (responders) compared to those who did not (non-responders) at the 24-week follow-up. We identified ten metabolites as the principal suggestive features driving the separation between responders and non-responders. Among these, nine were enriched at baseline in non-responders, including three acylcarnitines, while one (cytidine) was enriched in responders. Cytidine is a nucleoside which is deaminated to uridine by cytidine deaminases to maintain the intracellular pyrimidine pool [32]. Cytidine deaminase activity is essential for DNA and RNA synthesis. Serum cytidine deaminase is increased in people with RA compared to those with osteoarthritis and is associated with disease activity [33]. Moreover, a type of cytidine deaminases, namely activation-induced cytidine deaminase, plays a pivotal role in somatic hypermutation and antibody class switching, and its expression in blood cells has been associated with rheumatoid factor and ACPA levels in RA [34]. Interestingly, while cytidine was not associated with baseline CDAI in our analysis, it was positively associated with treatment response. This suggests that cytidine may be involved in modulating therapeutic outcomes, rather than simply reflecting baseline inflammatory burdens.

The one-factor univariate analysis, combining the results from t-test and fold change analyses in a volcano plot, confirmed cytidine as up regulated in responders. Out of the remaining six metabolites, two acylcarnitines were confirmed to be upregulated in non-responders together with malic acid and acetaminophen. Malic acid is an organic dicarboxylic acid that, in humans, is obtained through dietary intake and also synthesized through the citric acid cycle [35]. Malic acid plays a role in malate-aspartate shuttle that is responsible for the transportation of electrons produced during glycolysis through the mitochondria inner membrane to support oxidative phosphorylation [36]. While no prior study has specifically investigated malic acid in the context of RA, a previous work has suggested a potential involvement of the malate-aspartate shuttle in RA, albeit not linked to a treatment response [37].

The multivariable analysis adjusted for several covariates confirmed malic acid and cytidine as altered at baseline between responders and non-responders. Conversely, the association between acylcarnitines and response to treatment was lost after accounting for confounders such as baseline DAS28-CRP which suggests that acylcarnitines are highly associated with disease activity at baseline rather than future remission. The multivariable analysis also highlighted changes in other metabolites including uric acid, citrulline and L-arginine. Both citrulline and arginine are α-amino acids. One of the anabolic ways to produce citrulline is through arginine, but citrulline can be also converted to arginine [38]. Citrullination is a post-transcriptional protein modification consisting in the conversion of arginine to citrulline and is strongly linked to inflammation [39]. The production of autoantibodies against those citrullinated proteins, namely ACPA, is a hallmark of RA and is associated with bad prognosis and worse response to treatment [3, 40]. Although citrullinated proteins are associated with disease progression in RA, we observed positive association of citrulline with response. This could reflect an upregulated urea cycle or increased arginine metabolism rather than autoantigen presence. Elevated serum levels of L-arginine have been reported in people with established RA and at-risk individuals, who subsequently developed RA, compared to healthy controls [41, 42]. Moreover, people with RA who achieved sustained drug-free remission following treatment with methotrexate showed higher circulating arginine and proline concentrations [18]. Results from these studies are consistent with findings in our report of elevated levels of L-arginine in participants with RA who achieved CDAI remission.

A pathway enrichment analysis of our data further confirmed alterations in several metabolic pathways in responders compared to non-responders. Notably, these included arginine and proline metabolism, carnitine synthesis, and the urea cycle. Both L-arginine and citrulline are components of the urea cycle. Previous studies have also shown an upregulation of urea cycle in treatment-naïve RA participants [19] along with its strong association with higher fatigue scores and muscle wasting in people with RA [43]. Moreover, levels of metabolites that are part of urea cycle i.e., aspartic acid, citrulline, and arginine, have been reported to be increased in people with RA compared to controls [44]. Given that only a limited number of metabolites met the criteria to run the pathway enrichment analysis, these results should be interpreted as exploratory. However, we believe it is important to present these findings to highlight the potential impact and biological relevance of individual metabolites within specific pathways, particularly in the context of our hypothesis-free study design.

One of the strengths of the current report is, together with the selection of participants with untreated early RA, the randomization at baseline in four treatment arms, ranging from conventional treatment with methotrexate and prednisolone to three different biological drugs combined to methotrexate. After stratifying the cohort in the four treatment arms, we repeated the univariate and multivariate analyses previously performed in the main cohort. However, this analysis is limited by the low number of participants in each arm (slightly above 50 each). The analysis after stratification by treatment arms confirmed that arginine and citrulline were increased in responders vs. non-responders in arm 1 (conventional treatment), arginine in arm 4 (tocilizumab), and that subsequently the urea cycle was altered. Similarly, a recent study has also highlighted elevated levels of metabolites that are part of the urea cycle in people with active RA who responded to tocilizumab therapy [13]. We also found that pathways involved in fatty acid and carnitines metabolism were associated with response to treatment in arm 1, 2 (certolizumab-pegol), and 3 (abatacept). Only one previous study has investigated changes in the metabolic profile in RA after treatment with TNF-inhibitors showing an increase of hippuric acid, citrate, and lactic acid related to infliximab use, while increases in choline, phenylacetic acid, urea, creatine, and methylamine were observed after treatment with etanercept [45].

Following our profiling of metabolic alterations in people with early RA in relation to disease activity and response to treatment, we developed prediction models based on baseline metabolites and clinical data to discriminate between responders and non-responders at 24 weeks using four machine learning algorithms. Metabolomics combined with machine learning has been previously used to build prediction model of RA diagnosis or of disease activity [11, 28]. Only one lipidomics-based model was developed to predict response to treatment, namely methotrexate, in RA, showing poor predictive value [46]. In our study, we identified 15 features as potential biomarkers for distinguishing between responder and non-responder groups, with an AUC ranging from 0.55 to 0.73 in the test dataset. We acknowledge that these prediction models are far from being applicable in a clinical setting. However, these models combining clinical and metabolic data showed a better potential to predict treatment response than the models created with clinical parameters only. Moreover, this does not undermine the biological relevance of these features in relation to treatment response in early RA. Previous studies employing machine learning prediction models on genomic data have consistently yielded lower predictive power for various complex disease outcomes [47, 48]. Nevertheless, these findings do not invalidate the biologically important role of these genes in relation to the specific clinical outcomes.

Our study is not without limitations. First, we analyzed the data of only pre-annotated metabolites, which might have resulted in missing some unknown, yet important, metabolites in connection to achieving remission in people with early RA. Second, while we did adjust our analyses for potential, and known, confounders that could influence treatment response in RA, the associations did not pass the FDR correction criteria and should only be deemed suggestive. Third, the analysis stratified by treatment arm may be constrained by low statistical power owing to the small sample size within each arm and should be interpreted with caution. Moreover, the machine learning modelling of selected variables in our report had limited prediction values. This can be partially attributed to the study sample size as well as to the heterogeneity of treatments received by the participants which makes the prediction of response challenging. We also acknowledge that in our study responders had significantly lower baseline CDAI scores, which may reflect a greater intrinsic likelihood of remission. This inherent difference raises the possibility that some of the observed associations between metabolic profiles and treatment response could, in part, reflect baseline disease severity rather than being purely predictive biomarkers of response. While our multivariable analyses adjusted for baseline clinical covariates including CDAI, we recognize that residual confounding cannot be entirely ruled out. This distinction is important when interpreting whether a given metabolite reflects a propensity to respond or simply lower disease severity at treatment start.

## Conclusions

In summary, by performing high performance MS-based metabolomics in serum samples of 220 people with newly diagnosed RA who received four different therapeutical treatments and were followed-up for 24 weeks after treatment, our study has provided novel insights into the metabolomic profile of people with early RA, with the identification of several metabolites associated with baseline disease activity and with remission at follow-up. Specifically, we have shown that several acylcarnitines and amino acids were inversely associated with disease activity at baseline, whereas circulating malic acid, arginine, citrulline, and cytidine levels and their metabolic pathways were associated with CDAI remission at the 24-week follow-up. Given the exploratory nature of our study, these associations should be interpreted as hypothesis-generating rather than confirmatory, particularly as they did not pass multiple testing correction. Nevertheless, these findings provide valuable insights into metabolic alterations in early RA and their potential links to disease activity and treatment response. We also developed machine learning models integrating metabolomic and clinical features, which although showing a limited predictive value in distinguishing responders from non-responders in early RA, performed better than models including only clinical parameters. This highlights the potential of metabolomics in refining precision medicine approaches in RA, even though further validation is needed. As our study was hypothesis-free, we emphasize that the importance of the identified metabolites should not be assessed only based on their predictive value but also on their biological relevance, specifically their roles in metabolic and inflammatory pathways involved in RA pathophysiology. Future studies should aim to validate these findings in larger cohorts, and integrate metabolomics with other omics data (e.g., genomics and proteomics) for more robust models.

## Electronic supplementary material

Below is the link to the electronic supplementary material.

Supplementary Material 1

## Data Availability

No datasets were generated or analysed during the current study.

## Abbreviations

ACPA: Anti-citrullinated peptide antibody
AUC: Area under the receiver operating characteristic curve
BMI: Body mass index
CDAI: Clinical disease activity index
CRP: C-reactive protein
DNA: Deoxyribonucleic acid
ESR: Erythrocyte sedimentation rate
FC: Fold change
FDR: False discovery rate
GLM: Generalized linear model
HMBD: Human metabolome database
LC-MS: Liquid chromatography-mass spectrometry
MUVR: Multivariate methods with unbiased variable selection in R
NORD-STAR: Nordic rheumatic diseases strategy trials and registries
OPLS-DA: Orthogonal partial least squares discriminant analysis
PCA: Principal component analysis
PPC: Probabilistic principal component analysis
QC: Quality control
RA: Rheumatoid arthritis
RF: Rheumatoid factor
RNA: Ribonucleic acid
ROC: Receiver operating characteristic
SMPDB: The small molecule pathway database
SVM: Support vector machine
TNF: Tumor necrosis factor
UHPLC-QTOF: Ultra-high performance liquid chromatography-quadrupole time-of-flight mass spectrometry
VIP: Variable importance in projection
XGB: Extreme gradient boosting
DAS28-CRP: Disease activity score of 28 joints-based on C-reactive protein
DAS28-ESR: Disease activity score of 28 joints-based on erythrocyte sedimentation rate

## References

[1] Smolen J, Aletaha D, Barton A, Burmester G, Emery P, Firestein G. Rheumatoid arthritis. Nat Rev Dis Primers. 2018;4:18001. 29417936 10.1038/nrdp.2018.1

[2] Almutairi K, Nossent J, Preen D, Keen H, Inderjeeth C. The global prevalence of rheumatoid arthritis: a meta-analysis based on a systematic review. Rheumatol Int. 2021;41(5):863–77. 33175207 10.1007/s00296-020-04731-0

[3] Smolen JS, Landewé RB, Bijlsma JW, Burmester GR, Dougados M, Kerschbaumer A, McInnes IB, Sepriano A, Van Vollenhoven RF, De Wit M. EULAR recommendations for the management of rheumatoid arthritis with synthetic and biological disease-modifying antirheumatic drugs: 2019 update. Ann Rheum Dis. 2020;79(6):685–99. 31969328 10.1136/annrheumdis-2019-216655

[4] Hetland ML, Haavardsholm EA, Rudin A, Nordström D, Nurmohamed M, Gudbjornsson B, Lampa J, Hørslev-Petersen K, Uhlig T, Grondal G. Active conventional treatment and three different biological treatments in early rheumatoid arthritis: phase IV investigator initiated, randomised, observer blinded clinical trial. BMJ 2020;371:m4328. 10.1136/bmj.m4328 PMC7708829 33268527

[5] Finckh A, Gilbert B, Hodkinson B, Bae S-C, Thomas R, Deane KD, Alpizar-Rodriguez D, Lauper K. Global epidemiology of rheumatoid arthritis. Nat Rev Rheumatol. 2022;18(10):591–602. 36068354 10.1038/s41584-022-00827-y

[6] Watanabe R, Okano T, Gon T, Yoshida N, Fukumoto K, Yamada S, Hashimoto M. Difficult-to-treat rheumatoid arthritis: current concept and unsolved problems. Front Med. 2022;9:1049875. 10.3389/fmed.2022.1049875 PMC9637686 36353219

[7] Beger RD, Dunn W, Schmidt MA, Gross SS, Kirwan JA, Cascante M, Brennan L, Wishart DS, Oresic M, Hankemeier T. Metabolomics enables precision medicine:a white paper, community perspective. Metabolomics. 2016;12:1–15. 10.1007/s11306-016-1094-6 PMC5009152 27642271

[8] Rootwelt H, Elgstøen KBP. Metabolomics–a new biochemical golden age for personalised medicine. Tidsskrift Den Norske Legeforening 2022:142. 10.4045/tidsskr.22.0034 35383452

[9] Shen X, Wang C, Liang N, Liu Z, Li X, Zhu ZJ, Merriman TR, Dalbeth N, Terkeltaub R, Li C. Serum metabolomics identifies dysregulated pathways and potential metabolic biomarkers for hyperuricemia and gout. Arthritis Rheumatol. 2021;73(9):1738–48. 33760368 10.1002/art.41733

[10] Buergel T, Steinfeldt J, Ruyoga G, Pietzner M, Bizzarri D, Vojinovic D, Upmeier zu Belzen J, Loock L, Kittner P, Christmann L. Metabolomic profiles predict individual multidisease outcomes. Nat Med. 2022;28(11):2309–20. 36138150 10.1038/s41591-022-01980-3 PMC9671812

[11] Luan H, Gu W, Li H, Wang Z, Lu L, Ke M, Lu J, Chen W, Lan Z, Xiao Y. Serum metabolomic and lipidomic profiling identifies diagnostic biomarkers for seropositive and seronegative rheumatoid arthritis patients. J Translational Med. 2021;19(1):1–10. 10.1186/s12967-021-03169-7 PMC8650414 34876179

[12] Cedeno M, Murillo-Saich J, Coras R, Cedola F, Brandy A, Prior A, Pedersen A, Mateo L, Martinez-Morillo M, Guma M. Serum metabolomic profiling identifies potential biomarkers in arthritis in older adults: an exploratory study. Metabolomics. 2023;19(4):37. 37022535 10.1007/s11306-023-02004-y PMC11449491

[13] Murillo-Saich JD, Diaz-Torne C, Ortiz MA, Coras R, Gil-Alabarse P, Pedersen A, Corominas H, Vidal S, Guma M. Metabolomics profiling predicts outcome of Tocilizumab in rheumatoid arthritis: an exploratory study. Metabolomics. 2021;17(9):74. 34402961 10.1007/s11306-021-01822-2 PMC8810395

[14] Medcalf MR, Bhadbhade P, Mikuls TR, O’dell JR, Gundry RL, Funk RS. Plasma metabolome normalization in rheumatoid arthritis following initiation of methotrexate and the identification of metabolic biomarkers of efficacy. Metabolites. 2021;11(12):824. 34940582 10.3390/metabo11120824 PMC8706490

[15] Macáková K, Tekeľová M, Mlynáriková V, Šebeková K, Vlková B, Celec P, Šteňová E. Metabolic effects of Anti-TNF-α treatment in rheumatoid arthritis. Diseases. 2023;11(4):164. 37987275 10.3390/diseases11040164 PMC10660495

[16] Sasaki C, Hiraishi T, Oku T, Okuma K, Suzumura K, Hashimoto M, Ito H, Aramori I, Hirayama Y. Metabolomic approach to the exploration of biomarkers associated with disease activity in rheumatoid arthritis. PLoS ONE. 2019;14(7):e0219400. 31295280 10.1371/journal.pone.0219400 PMC6622493

[17] Young SP, Kapoor SR, Viant MR, Byrne JJ, Filer A, Buckley CD, Kitas GD, Raza K. The impact of inflammation on metabolomic profiles in patients with arthritis. Arthr Rhuem. 2013;65(8):2015–23. 10.1002/art.38021 PMC3840700 23740368

[18] Teitsma XM, Yang W, Jacobs JW, Pethö-Schramm A, Borm ME, Harms AC, Hankemeier T, van Laar JM, Bijlsma JW, Lafeber FP. Baseline metabolic profiles of early rheumatoid arthritis patients achieving sustained drug-free remission after initiating treat-to-target tocilizumab, methotrexate, or the combination: insights from systems biology. Arthritis Res Therapy. 2018;20:1–11. 10.1186/s13075-018-1729-2 PMC6235217 30322408

[19] Jutley GS, Sahota K, Sahbudin I, Filer A, Arayssi T, Young SP, Raza K. Relationship between inflammation and metabolism in patients with newly presenting rheumatoid arthritis. Front Immunol. 2021;12:676105. 34650548 10.3389/fimmu.2021.676105 PMC8507469

[20] Østergaard M, Van Vollenhoven RF, Rudin A, Hetland ML, Heiberg MS, Nordström DC, Nurmohamed MT, Gudbjornsson B, Ørnbjerg LM, Bøyesen P. Certolizumab pegol, abatacept, Tocilizumab or active conventional treatment in early rheumatoid arthritis: 48-week clinical and radiographic results of the investigator-initiated randomised controlled NORD-STAR trial. Ann Rheum Dis. 2023;82(10):1286–95. 37423647 10.1136/ard-2023-224116

[21] Xia J, Wishart DS. Metabolomic data processing, analysis, and interpretation using MetaboAnalyst. Current protocols in bioinformatics 2011;34(1):14–10. 10.1002/0471250953.bi1410s34 21633943

[22] Team RC. R: A language and environment for statistical computing. R Foundation for Statistical Computing, Vienna, Austria 2021. https://www.R-project.org/

[23] Triba MN, Le Moyec L, Amathieu R, Goossens C, Bouchemal N, Nahon P, Rutledge DN, Savarin P. PLS/OPLS models in metabolomics: the impact of permutation of dataset rows on the K-fold cross-validation quality parameters. Mol Biosyst. 2015;11(1):13–9. 25382277 10.1039/c4mb00414k

[24] Chong I-G, Jun C-H. Performance of some variable selection methods when multicollinearity is present. Chemometr Intell Lab Syst. 2005;78(1–2):103–12.

[25] Nyamundanda G, Gormley IC, Fan Y, Gallagher WM, Brennan L. MetSizeR: selecting the optimal sample size for metabolomic studies using an analysis based approach. BMC Bioinformatics. 2013;14(1):1–8. 24261687 10.1186/1471-2105-14-338 PMC4222287

[26] Shi L, Westerhuis JA, Rosén J, Landberg R, Brunius C. Variable selection and validation in multivariate modelling. Bioinformatics. 2019;35(6):972–80. 30165467 10.1093/bioinformatics/bty710 PMC6419897

[27] Dambrova M, Makrecka-Kuka M, Kuka J, Vilskersts R, Nordberg D, Attwood MM, Smesny S, Sen ZD, Guo AC, Oler E. Acylcarnitines: nomenclature, biomarkers, therapeutic potential, drug targets, and clinical trials. Pharmacol Rev. 2022;74(3):506–51. 35710135 10.1124/pharmrev.121.000408

[28] Hur B, Gupta VK, Huang H, Wright KA, Warrington KJ, Taneja V, Davis JM III, Sung J. Plasma metabolomic profiling in patients with rheumatoid arthritis identifies biochemical features predictive of quantitative disease activity. Arthritis Res Therapy. 2021;23(1):164. 10.1186/s13075-021-02537-4 PMC8185925 34103083

[29] Huffman KM, Jessee R, Andonian B, Davis BN, Narowski R, Huebner JL, Kraus VB, McCracken J, Gilmore BF, Tune KN. Molecular alterations in skeletal muscle in rheumatoid arthritis are related to disease activity, physical inactivity, and disability. Arthritis Res Therapy. 2017;19:1–17. 10.1186/s13075-016-1215-7 PMC5260091 28114971

[30] Chu SH, Cui J, Sparks JA, Lu B, Tedeschi SK, Speyer CB, Moss L, Feser ML, Kelmenson LB, Mewshaw EA. Circulating plasma metabolites and risk of rheumatoid arthritis in the nurses’ health study. Rheumatology. 2020;59(11):3369–79. 32310291 10.1093/rheumatology/keaa125 PMC7590418

[31] de Jong T, Semmelink J, Denis S, van de Sande M, Houtkooper R, van Baarsen L. Altered lipid metabolism in synovial fibroblasts of individuals at risk of developing rheumatoid arthritis. J Autoimmun. 2023;134:102974. 36512907 10.1016/j.jaut.2022.102974

[32] Frances A, Cordelier P. The emerging role of cytidine deaminase in human diseases: a new opportunity for therapy? Mol Ther. 2020;28(2):357–66. 31870623 10.1016/j.ymthe.2019.11.026 PMC7001087

[33] Thompson P, Jones D, Currey H. Cytidine deaminase activity as a measure of acute inflammation in rheumatoid arthritis. Ann Rheum Dis. 1986;45(1):9. 3954467 10.1136/ard.45.1.9 PMC1001807

[34] Xu X, Hsu HC, Chen J, Grizzle WE, Chatham WW, Stockard CR, Wu Q, Yang P, Holers VM, Mountz JD. Increased expression of activation-induced cytidine deaminase is associated with anti‐CCP and rheumatoid factor in rheumatoid arthritis. Scand J Immunol. 2009;70(3):309–16. 19703021 10.1111/j.1365-3083.2009.02302.x PMC3674772

[35] Wishart DS, Guo A, Oler E, Wang F, Anjum A, Peters H, Dizon R, Sayeeda Z, Tian S, Lee BL. HMDB 5.0: the human metabolome database for 2022. Nucleic Acids Res. 2022;50(D1):D622–31. 34986597 10.1093/nar/gkab1062 PMC8728138

[36] Dawson A. Oxidation of cytosolic NADH formed during aerobic metabolism in mammalian cells. Trends Biochem Sci. 1979;4(8):171–6.

[37] Wu B, Zhao TV, Jin K, Hu Z, Abdel MP, Warrington KJ, Goronzy JJ, Weyand CM. Mitochondrial aspartate regulates TNF biogenesis and autoimmune tissue inflammation. Nat Immunol. 2021;22(12):1551–62. 34811544 10.1038/s41590-021-01065-2 PMC8756813

[38] Osowska S, Moinard C, Loï C, Neveux N, Cynober L. Citrulline increases arginine pools and restores nitrogen balance after massive intestinal resection. Gut. 2004;53(12):1781. 15542514 10.1136/gut.2004.042317 PMC1774314

[39] Makrygiannakis D, af Klint E, Lundberg IE, Löfberg R, Ulfgren AK, Klareskog L, Catrina AI. Citrullination is an inflammation dependent process. Annals of the rheumatic diseases 2006;65:1219–1222. 10.1136/ard.2005.049403 PMC1798285 16540548

[40] Seegobin SD, Ma MH, Dahanayake C, Cope AP, Scott DL, Lewis CM, Scott IC. ACPA-positive and ACPA-negative rheumatoid arthritis differ in their requirements for combination DMARDs and corticosteroids: secondary analysis of a randomized controlled trial. Arthritis Res Therapy. 2014;16:1–12. 10.1186/ar4439 PMC3979097 24433430

[41] Huang L-W, Chang K-L, Chen C-J, Liu H-W. Arginase levels are increased in patients with rheumatoid arthritis. Kaohsiung J Med Sci. 2001;17(7):358–63. 11593962

[42] Cao S, Li Y, Song R, Meng X, Fuchs M, Liang C, Kachler K, Meng X, Wen J, Schlötzer-Schrehardt U. L-arginine metabolism inhibits arthritis and inflammatory bone loss. Ann Rheum Dis. 2024;83(1):72–87. 37775153 10.1136/ard-2022-223626 PMC10803985

[43] Bartikoski BJ, De Oliveira MS, do, Espírito Santo RC, Dos Santos LP, Dos Santos NG, Xavier RM. A review of metabolomic profiling in rheumatoid arthritis: bringing new insights in disease pathogenesis, treatment and comorbidities. Metabolites. 2022;12(5):394. 10.3390/metabo12050394 PMC9146149 35629898

[44] Xu L, Chang C, Jiang P, Wei K, Zhang R, Jin Y, Zhao J, Xu L, Shi Y, Guo S. Metabolomics in rheumatoid arthritis: advances and review. Front Immunol. 2022;13:961708. 36032122 10.3389/fimmu.2022.961708 PMC9404373

[45] Priori R, Casadei L, Valerio M, Scrivo R, Valesini G, Manetti C. 1H-NMR-based metabolomic study for identifying serum profiles associated with the response to etanercept in patients with rheumatoid arthritis. PLoS ONE. 2015;10(11):e0138537. 26558759 10.1371/journal.pone.0138537 PMC4641599

[46] Maciejewski M, Sands C, Nair N, Ling S, Verstappen S, Hyrich K, Barton A, Ziemek D, Lewis MR, Plant D. Prediction of response of methotrexate in patients with rheumatoid arthritis using serum lipidomics. Sci Rep. 2021;11(1):7266. 33790392 10.1038/s41598-021-86729-7 PMC8012618

[47] Lello L, Raben TG, Yong SY, Tellier LC, Hsu SD. Genomic prediction of 16 complex disease risks including heart attack, diabetes, breast and prostate cancer. Sci Rep. 2019;9(1):15286. 31653892 10.1038/s41598-019-51258-x PMC6814833

[48] Widen E, Raben TG, Lello L, Hsu SD. Machine learning prediction of biomarkers from SNPs and of disease risk from biomarkers in the UK biobank. Genes. 2021;12(7):991. 34209487 10.3390/genes12070991 PMC8308062

