# Supplementary material for: Disease activity and treatment response in early rheumatoid arthritis: an exploratory metabolomic profiling in the NORD-STAR cohort
Source: Arthritis Res Ther. 2025 Jul 26;27:156. doi: 10.1186/s13075-025-03616-6 (PMC12297794; doi:10.1186/s13075-025-03616-6)
Supplement: Supplementary file 1 — Supplementary Material 1 [file 13075_2025_3616_MOESM1_ESM.pdf]

1 Supplementary text

MS metabolomics profiling and analysis

Sample Preparation

Sample preparation of serum samples was performed with a semi-automated approach using an Agilent Bravo automatic liquid handling platform (G5563AA, Agilent Technologies, CA, USA) and the accompanying software Agilent VWorks for automation control. 720 µl of extraction buffer (90/10 v/v methanol: water) including internal standards (see supplementary) was added to extract plates (96 Agilent 203426-100 PP, 1 mL Rnd Btm). 80 µl of the serum samples were added to the extract plates and the plates were mixed using the Bravo system (at 1 000 rpm with 10 mixes). The proteins were precipitated at -20°C for 2 hours. The sample plates were centrifuged at 4°C, 4000 rpm (3 488 g), for 10 minutes on a tabletop swingout rotor. 200µL supernatant was transferred to LC (liquid chromatography) plates (96 Agilent WP 5043-9314 0.33 mL VBtm) using the Bravo system. The sample plates were evaporated to dryness under a stream of nitrogen (30L min-1 at 30°C) and stored at -80°C until analysis. Small aliquots of the remaining supernatants were pooled and used to create quality control (QC) samples.

UHPLC-Q-TOF-MS analysis

Before analysis, the samples were re-suspended in 10+10 µL methanol and water. The samples were analyzed in batches according to a randomized run order (randomized by treatment arms and sex). Each batch of samples was first analyzed in positive mode. After all samples within a batch had been analyzed, the instrument was switched to negative mode and a second injection of each sample was performed.

The chromatographic separation was performed on an Agilent 1290 Infinity UHPLC-system (Agilent Technologies, Waldbronn, Germany). 2 µL of each sample were injected onto an Acquity UPLC HSS T3, 2.1 x 50 mm, 1.8 µm C18 column in combination with a 2.1 mm x 5 mm, 1.8 µm VanGuard precolumn (Waters Corporation, Milford, MA, USA) held at 40°C. The gradient elution buffers were A (H_2O, 0.1 % formic acid) and B (75/25 acetonitrile:2-propanol, 0.1 % formic acid), and the flow-rate was 0.5 mL min-1. The compounds were eluted with a

linear gradient consisting of 0.1 - 10 % B over 2 minutes, B was increased to 99 % over 5 minutes and held at 99 % for 2 minutes; B was decreased to 0.1 % for 0.3 minutes and the flow-rate was increased to 0.8 mL min-1 for 0.5 minutes; these conditions were held for 0.9 minutes, after which the flow-rate was reduced to 0.5 mL min-1 for 0.1 minutes before the next injection.

The compounds were detected with an Agilent 6546 Q-TOF (quadrupole time-of-flight) mass spectrometer (MS) equipped with a jet stream electrospray ion source operating in positive or negative ion mode. The settings were kept identical between the modes, with the exception of the capillary voltage. A reference interface was connected for accurate mass measurements; the reference ions purine (4 μM) and HP-0921 (Hexakis(1H, 1H, 3H-tetrafluoropropoxy)phosphazine) (1 μM) were infused directly into the MS at a flow rate of 0.05 mL min-1 for internal calibration, and the monitored ions were purine m/z 121.05 and m/z 119.03632; HP-0921 m/z 922.0098 and m/z 966.000725 for positive and negative mode respectively. The gas temperature was set to 150°C, the drying gas flow to 8 L min-1 and the nebulizer pressure 35 psig. The sheath gas temp was set to 350°C and the sheath gas flow 11 L min-1. The capillary voltage was set to 4000 V in positive ion mode, and to 4000 V in negative ion mode. The nozzle voltage was 300 V. The fragmentor voltage was 120 V, the skimmer 65 V and the OCT 1 RF Vpp 750 V. The collision energy was set to 0 V. The m/z range was 70 - 1700, and data was collected in centroid mode with an acquisition rate of 4 scans s-1 (1977 transients/spectrum). MSMS analysis was run on the QC samples for identification purposes.

Data pre-processing

All data pre-processing was carried out using Agilent MassHunter Profinder (version B.10.0 SP1, Agilent Technologies Inc., Santa Clara, CA, USA), employing a targeted data pre-processing approach. The term "targeted pre-processing approach" refers to a focused data pre-processing strategy, where a predefined list of metabolites commonly found in plasma and serum was actively searched rather than performing an untargeted, peak picking approach) using the Batch Targeted Feature Extraction function. This targeted approach utilizes a compound-specific library, which includes retention times (RT) and elemental compositions. More specifically, the SMC in-house LC-MS library, constructed from authentic standards analyzed using the same system and chromatographic/mass spectrometric settings, served as the reference panel. This library, comprising over 700 compounds, enabled the confident

annotation of 278 metabolites Annotation was carried out based on a combination of MS and MS/MS spectral data and retention times. To ensure high data quality, all detected metabolite peaks were manually verified, and their integration curated across all samples using the overlay function, applying consistent RT intervals. This practice significantly minimized technical variability and reduced the prevalence of missing values across the dataset. However, in instances where a metabolite was still undetected in a specific sample (despite targeted integration), a conservative imputation strategy was applied. Specifically, missing values were imputed as 0.9 times the minimum detected value for that particular metabolite. This approach is commonly used to approximate low abundance values without introducing artificial variation, thereby preserving the integrity of downstream statistical analyses.

Information about reagents, solvents, standards, reference and tuning standards, and stable isotopes internal standards

Solvents

Methanol, HPLC-grade was obtained from Fischer Scientific (Waltham, MA, USA) Acetonitrile, LC-MS grade was obtained from Merck (Kenilworth, NJ, USA)2-Propanol, LC-MS grade was obtained from Merck (Kenilworth, NJ, USA)
H2O, Milli-Q.

Reference and tuning standards

Purine, 4 µM, Agilent Technologies (Santa Clara, CA, USA) HP-0921 (Hexakis (1H, 1H, 3H-tetrafluoropropoxy) phosphazine), 1 µM, Agilent Technologies (Santa Clara, CA, USA) Calibrant, ESI-TOF, ESI-L Low Concentration Tuning Mix, Agilent Technologies (Santa Clara, CA, USA) HP-0321 (Hexamethoxyphosphazine), 0.1 mM, Agilent Technologies (Santa Clara, CA, USA).

Stable isotopes internal standard

13C9-Phenylalanine, 13C3-Caffeine, D4-Cholic acid, 13C9-Caffeic Acid and Salicylic acid-D6 were obtained from Sigma (St. Louis, MO, USA).

Reference

1. A J, Trygg J, Gullberg J, Johansson AI, Jonsson P, Antti H, Marklund SL & Moritz T. Extraction and GC/MS analysis of the human blood plasma metabolome. *Anal Chem* 2005 **77** 8086-8094.

2 Supplementary tables and figures

Table S1 Comparison of the subset (n = 220) from NORDSTAR cohort (n=393) selected for metabolomics profiling with rest of the cohort (n = 173)

Variables	Included in metabolomics		*p*-value
	yes	no	
**Baseline**			
N	220	173	-
Female, n (%)	153 (69)	117 (67)	0.68
Age, years	54 ± 15	57 ± 15	0.14
BMI	26 ± 5	27 ± 5	0.28
Current smokers, n (%)	44 (20)	37 (21)	0.73
RF positive, n (%)	162 (73)	132 (76)	0.48
ACPA positive, n (%)	180 (81)	138 (79)	0.60
Symptom duration, days	215 ± 169	194 ± 151	0.20
Time since diagnosis, days	8 ± 26	9 ± 15	0.72
ESR, mm/h	34 ± 25	35 ± 26	0.75
CRP, mg/L	22 ± 31	23 ± 29	0.93
SJC28	8 ± 5	9 ± 6	0.08
TJC28	10 ± 6	10 ± 6	0.67
DAS28-ESR	5.5 ± 1.1	5.6 ± 1.1	0.57
DAS28-CRP	5.1 ± 1.1	5.2 ± 1.0	0.57
CDAI	29 ± 12	31 ± 12	0.19
**24 weeks**			
ESR, mm/h	11 ± 11	13 ± 13	0.15
CRP, mg/L	2.5 ± 4.2	2.8 ± 4.2	0.50
SJC28	1 ± 1	1 ± 2	0.94
TJC28	2 ± 3	2 ± 3	0.69
DAS28-ESR	2.3 ± 1.2	2.3 ± 1.1	0.66
DAS28-CRP	2.2 ± 0.9	2.2 ± 0.9	0.81
CDAI	5.5 ± 5.6	5.4 ± 5.6	0.85

Continuous variables are expressed as mean ± standard deviation, while categorical variables are expressed as number and percentages. Student t-test was used to compare continuous variables while Chi-square test was used to compare categorical variables between responders and non-responders' groups and *p*-values are provided for a comparison between these two groups.

N: Number of people with early RA, BMI: body mass index, RF: rheumatoid factor, ACPA: anti-citrullinated peptide antibody, ESR: Erythrocyte sedimentation rate, CRP: C-reactive protein, SJC28: Swollen joint count (out of 28), TJC28: Tender joint count (out of 28), DAS28-ESR: Disease activity score of 28 joints (ESR-based), DAS28-CRP: Disease activity score using 28 joint counts (CRP-based), CDAI: Clinical Disease Activity Index.

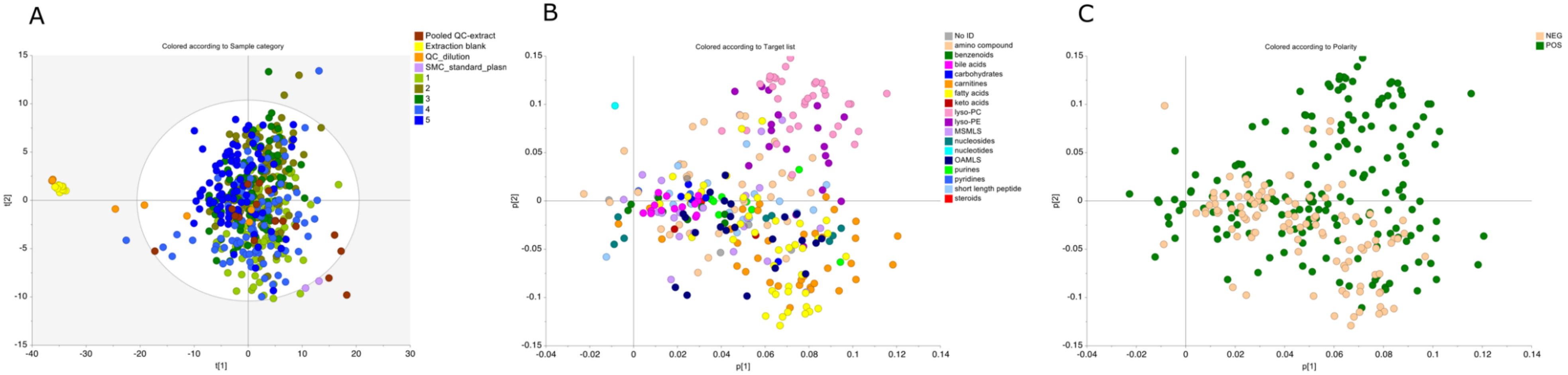

Figure S1 Loading plots from the principal component analysis (PCA) of the LC-MS illustrating a good data quality. The plots show stratification based on A) sample/batch category, B) metabolite class and C) ionization mode.
QC: quality controls, NEG: negative ionization mode, POS: positive ionization mode.

Table S2 Results from regression analysis to assess the association of baseline metabolites with baseline CDAI

Metabolite	Unadjusted		Adjusted†	
	Estimate	*p*-value	Estimate	*p*-value
Quinic acid	-2.608	0.009	-3.757	0.0009
Theophylline	-3.521	0.005	-4.321	0.0009
LysoPC(15:0/0:0)	-5.877	0.006	-6.63	0.002
N-gamma-Glutamylglutamine	-6.449	0.004	-6.675	0.003
Paraxanthine	-2.834	0.016	-3.547	0.004
2-Methylbutyroylcarnitine	-3.456	0.016	-4.251	0.004
LysoPC(0:0/17:0)	-4.982	0.012	-5.679	0.005
3-Hydroxybutyrylcarnitine	-3.071	0.006	-3.2	0.005
2-Hydroxydecanoate	-4.509	0.007	-4.719	0.006
LysoPC(17:0/0:0)	-5.18	0.011	-5.737	0.006
Lenticin	-2.768	0.008	-2.896	0.008
Caffeine	-2.368	0.026	-2.952	0.008
LysoPC(0:0/18:2(9Z,12Z))	-5.322	0.008	-5.421	0.008
LysoPC(0:0/15:0)	-4.533	0.015	-4.953	0.009
Citric acid	-4.519	0.019	-5.128	0.009
gamma-Aminobutyric acid	-5.172	0.014	-5.432	0.011
3-Hydroxytetradecanoic acid	-5.216	0.017	-5.599	0.011
LysoPC(18:2(9Z,12Z)/0:0)	-6.051	0.014	-6.351	0.012
LysoPC(20:1(11Z)/0:0)	-5.173	0.02	-5.502	0.016
Tiglylcarnitine	-3.88	0.031	-4.51	0.017
Isovalerylcarnitine	-4.203	0.028	-4.892	0.017
Valerylcarnitine	-3.954	0.022	-4.295	0.019
L-Methionine	-5.447	0.017	-5.305	0.021
gamma-Glutamylmethionine	-5.029	0.018	-4.899	0.023
Guanine	-5.423	0.02	-5.278	0.025
gamma-Glutamylalanine	-4.519	0.027	-4.564	0.026
Phenylalanylproline	-5.327	0.014	-5.073	0.027
L-Glutamine	-4.641	0.04	-5.088	0.027
Trigonelline	-2.098	0.103	-3.038	0.031
LysoPE(0:0/18:2(9Z,12Z))	-4.071	0.033	-4.168	0.031
LysoPC(0:0/18:3)	-4.019	0.038	-4.175	0.032
Kynurenic acid	-3.676	0.064	-4.385	0.034
Acetaminophen	1.652	0.03	1.649	0.034
4-Pyridoxic acid	-3.614	0.033	-3.624	0.035
Homo-L-arginine	-3.471	0.047	-3.81	0.037
Uracil	-4.724	0.038	-4.762	0.038
Threonic acid	-2.824	0.096	-3.61	0.042
(11Z)-Eicoseneoylcarnitine	-3.629	0.08	-4.419	0.042
3-Hydroxyoctanoic acid	-3.145	0.057	-3.314	0.047

Results are presented only for the metabolites (n = 39) that showed a significant association with baseline CDAI. †Values are adjusted for age, sex and body mass index (BMI). CDAI: Clinical Disease Activity Index

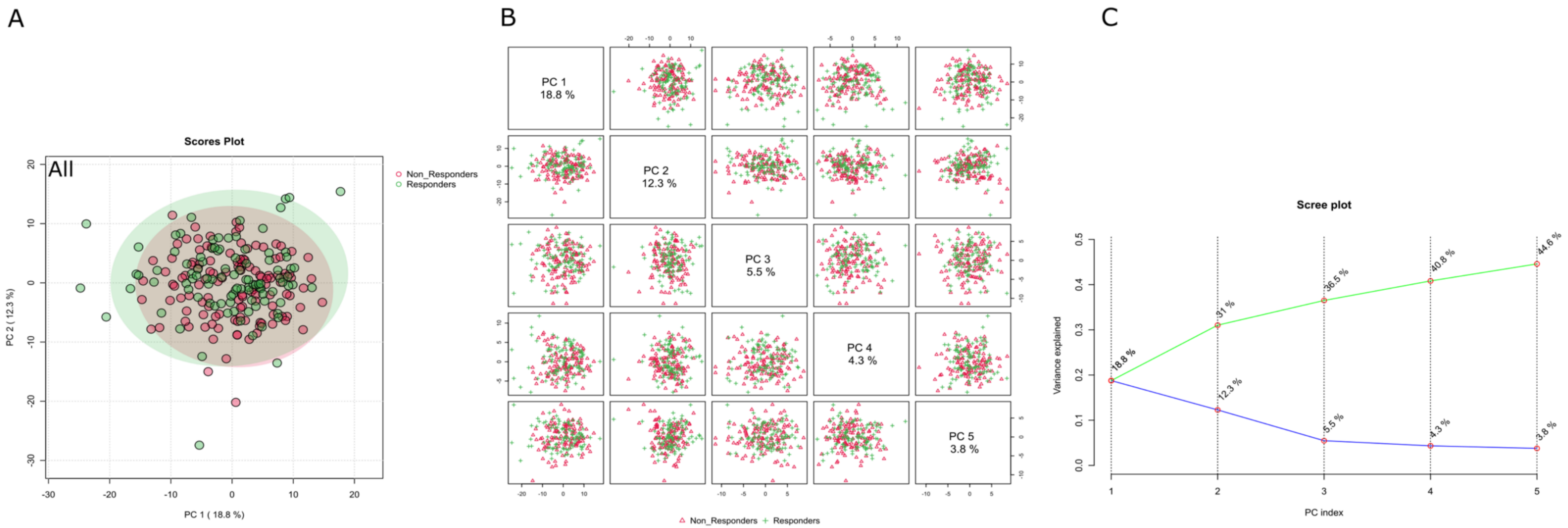

Figure S2 Results from principal component analysis (PCA) to A) indicate metabolite variation between responders and non-responders overall, B) pairwise plot showing the variance for components 1 to 5, C) Scree plot from PCA illustrating a total variance of 44.6%, explained via five components, between the two groups.

Table S3 Significant metabolites identified in one-factor analysis

**Metabolite name**	**FC**	***p*-value**	***p*-corrected**
Malic acid	0.802	0.004	0.354
Cytidine	1.271	0.006	0.354
3-Methylglutarylcarnitine	0.779	0.007	0.354
Butyrylcarnitine	0.813	0.009	0.354
2,4-Dihydroxybenzoic acid / 2-Pyrocatechuic acid	0.748	0.016	0.354
Acetaminophen	0.542	0.032	0.367
D-Phenyllactic acid	0.828	0.037	0.375

FC: Fold change, *p*-value: raw *p*-value, *p*-corrected: FDR corrected *p*-value

Note: A fold change of > 1.2 or < 0.83 and *p*-value (raw and corrected) ≤ 0.05 was used as a cut-off to indicate significance in fold change and t-test analyses, respectively.

Table S4 Results from primary multivariable (adjusted) analysis to assess the association of baseline metabolites with CDAI remission at 24 weeks in 220 participants included in the study

**Metabolite**	**Estimate [95% CI]**	***p*-value**	***p*-corrected**
Malic acid	-0.421 [-0.70 ; -0.14]	0.003	0.714
Cytidine	0.375 [0.10 ; 0.65]	0.008	0.714
Norvaline	0.358 [0.08 ; 0.64]	0.011	0.714
Uric acid	0.350 [0.07 ; 0.63]	0.013	0.714
L-Arginine	0.338 [0.06 ; 0.62]	0.017	0.714
gamma-Glutamylalanine	-0.320 [-0.60 ; -0.04]	0.024	0.714
L-Valine	0.317 [0.04 ; 0.60]	0.025	0.714
2,4-Dihydroxybenzoic acid / 2-Pyrocatechuic acid	-0.315 [-0.59 ; -0.04]	0.026	0.714
3-(3-Hydroxyphenyl)-3-hydroxypropanoic acid / 4-Hydroxyphenyllactic acid	-0.313 [-0.59 ; -0.04]	0.027	0.714
D-Glucoheptose	0.308 [0.03 ; 0.59]	0.030	0.714
LysoPC(0:0/17:0)	0.303 [0.03 ; 0.58]	0.032	0.714
Bis(2-ethylhexyl)phthalate	-0.301 [-0.58 ; -0.02]	0.034	0.714
Citrulline	0.299 [0.02 ; 0.58]	0.035	0.714
D-Phenyllactic acid	-0.290 [-0.57 ; -0.01]	0.041	0.714
LysoPC(0:0/18:0)	0.280 [0.00 ; 0.56]	0.048	0.714
4-Trimethylammoniobutanoic acid	0.279 [0.00 ; 0.56]	0.049	0.714
S-Adenosylhomocysteine	-0.279 [-0.56 ; 0.00]	0.049	0.714

95% CI: 95% confidence interval for regression estimate, *p*-value: raw *p*-value, *p*-corrected: FDR corrected *p*-value

Results are presented only for the metabolites (n = 17) that showed a significant association with CDAI remission at 24 weeks. Values are adjusted for age, sex, body mass index (BMI), anti-citrullinated protein antibody (ACPA) status, treatment randomization, current smoking status and DAS28-CRP: Disease activity score using 28 joint counts (CRP-based) at baseline. *p*-value (raw and corrected) ≤ 0.05 was used as a cut-off to indicate significance in the regression model.

CDAI: Clinical Disease Activity Index.

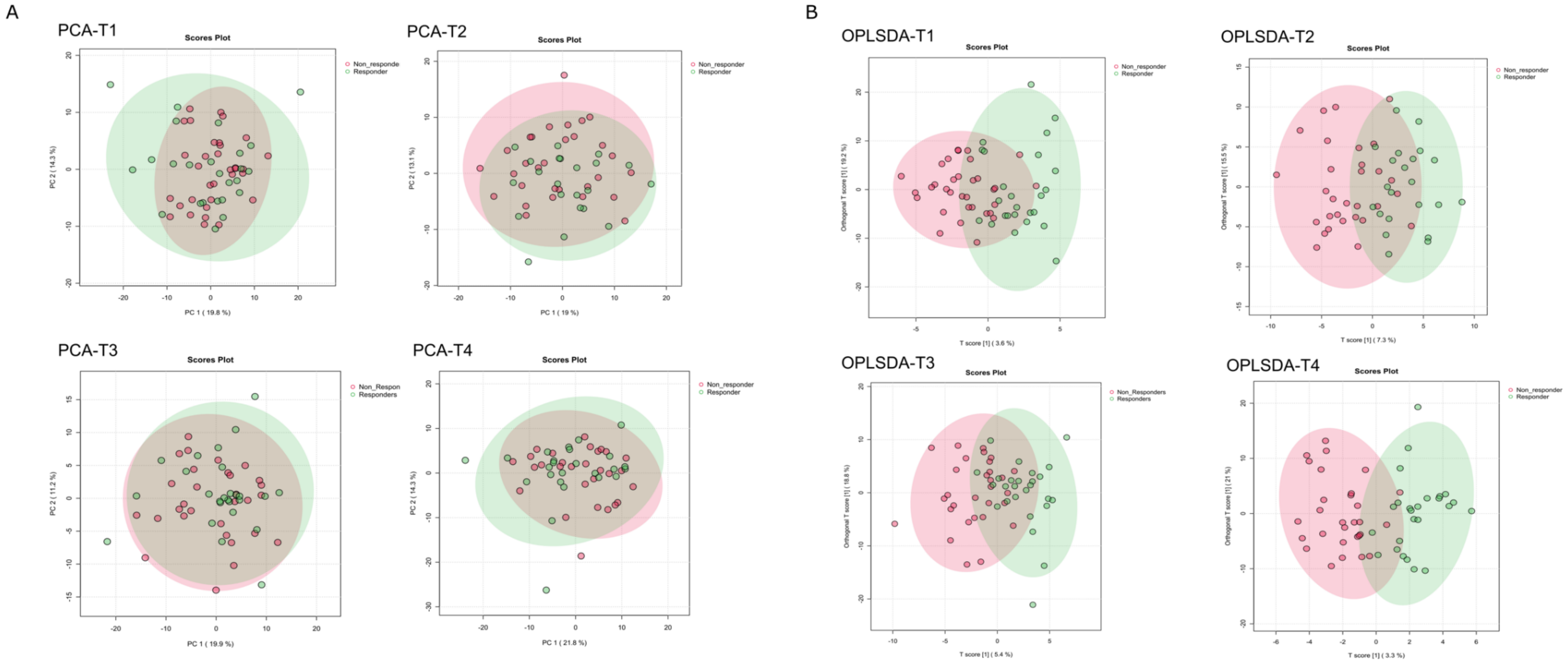

Figure S3 Score plots from A) principal component analysis (PCA) and B) orthogonal partial least squares discriminant analysis (OPLS-DA) to indicate metabolite variation to distinguish between responders and non-responders in four treatment (T1 to T4) groups, where 'T' refers to treatment arm.

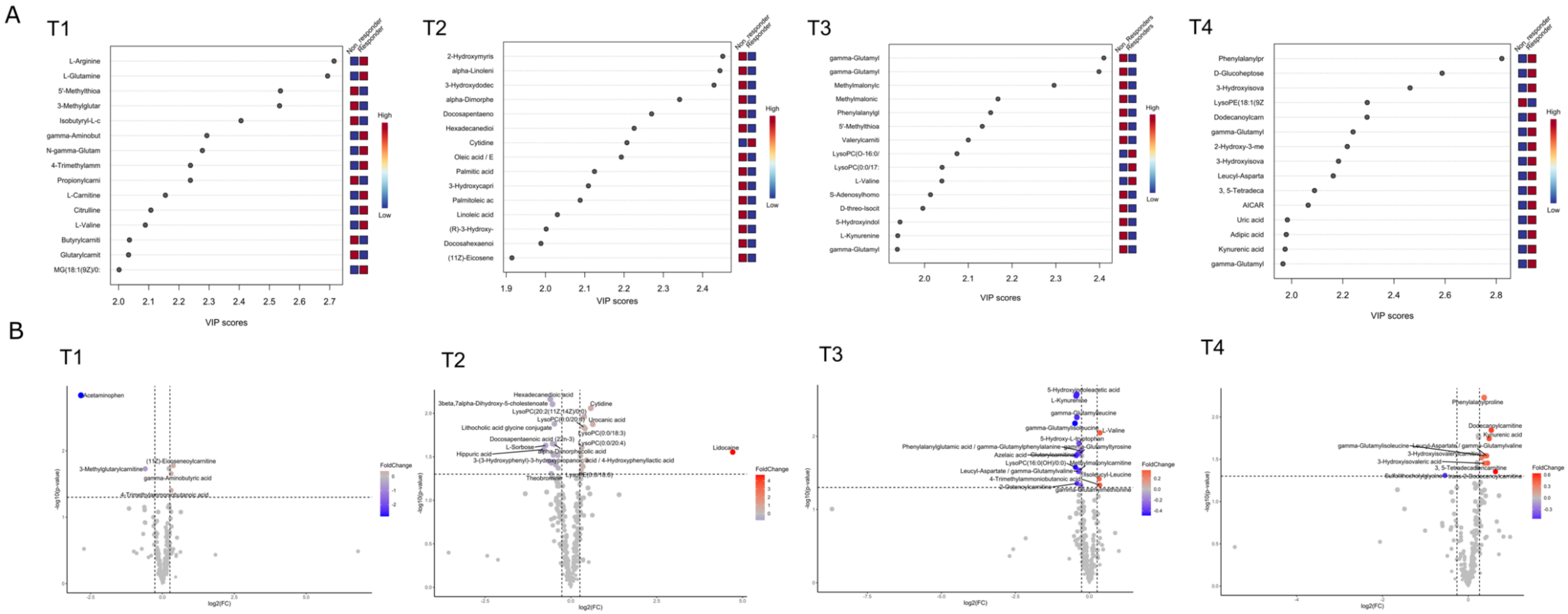

Figure S4 A) Variance of important projection (VIP) score plots of metabolites derived from orthogonal partial least squares discriminant analysis (OPLS-DA) illustrating the most important metabolites that discriminated responders from non-responders in four treatment (T1 to T4) groups, B) Volcano plot of differential metabolites obtained from one-factor univariate analyses in four treatment (T1 to T4) groups. Metabolites with a fold change of > 1.2 and a t-test $p \leq 0.05$ were considered significantly upregulated/enriched in responders, while metabolites with a fold change of < 0.83 and a t-test $p \leq 0.05$ were considered significantly upregulated/enriched in non-responders. 'T' refers to treatment arm.

Table S5 Results from primary multivariable (adjusted) analysis to assess the association of baseline metabolites with CDAI remission at 24 weeks in treatment 1 group (n = 58)

**Metabolite**	**Estimate [95% CI]**	***p*-value**	***p*-corrected**
L-Arginine	0.735 [0.15 ; 1.32]	0.014	0.998
Urocanic acid	-0.668 [-1.25 ; -0.08]	0.026	0.998
Acetoacetic acid	0.656 [0.07 ; 1.24]	0.028	0.998
4-Hydroxybenzoic acid	-0.655 [-1.24 ; -0.07]	0.029	0.998
Acetaminophen	-0.621 [-1.21 ; -0.03]	0.038	0.998
4-Hydroxyhippuric acid	-0.619 [-1.21 ; -0.03]	0.039	0.998
2,4-Dihydroxybenzoic acid / 2-Pyrocatechuic acid	-0.603 [-1.19 ; -0.02]	0.044	0.998
gamma-Aminobutyric acid	0.596 [0.01 ; 1.18]	0.046	0.998
Deoxycholic acid	-0.594 [-1.18 ; -0.01]	0.047	0.998

95% CI: 95% confidence interval for regression estimate, *p*-value: raw *p*-value, *p*-corrected: FDR corrected *p*-value

Results are presented only for the metabolites (n = 9) that showed a significant association with CDAI remission at 24 weeks in treatment 1 group. Values are adjusted for age, sex, body mass index (BMI), anti-citrullinated protein antibody (ACPA) status, treatment randomization, current smoking status and DAS28-CRP: Disease activity score using 28 joint counts (CRP-based) at baseline. CDAI: Clinical Disease Activity Index.

Table S6 Results from primary multivariable (adjusted) analysis to assess the association of baseline metabolites with CDAI remission at 24 weeks in treatment 2 group (n = 52)

Metabolite	Estimate [95% CI]	*p*-value	*p*-corrected
Hexadecanedioic acid	-0.840 [-1.41 ; -0.27]	0.004	0.484
Lidocaine	0.828 [0.26 ; 1.40]	0.004	0.484
2',4'-Dihydroxyacetophenone (Resorcinol monoacetate)	0.795 [0.22 ; 1.37]	0.006	0.484
alpha-Dimorphecolic acid	-0.738 [-1.31 ; -0.17]	0.011	0.484
3beta,7alpha-Dihydroxy-5-cholestenoate	-0.718 [-1.29 ; -0.15]	0.014	0.484
Cytidine	0.697 [0.13 ; 1.27]	0.017	0.484
3-Hydroxydodecanoic acid	-0.686 [-1.26 ; -0.11]	0.019	0.484
LysoPC(0:0/18:3)	0.678 [0.11 ; 1.25]	0.02	0.484
LysoPC(0:0/20:4)	0.673 [0.10 ; 1.24]	0.021	0.484
2-Hydroxymyristic acid	-0.669 [-1.24 ; -0.10]	0.022	0.484
3-(3-Hydroxyphenyl)-3-hydroxypropanoic acid / 4-Hydroxyphenyllactic acid	-0.666 [-1.24 ; -0.09]	0.022	0.484
Octadecanedioic acid	-0.656 [-1.23 ; -0.08]	0.024	0.484
Lithocholic acid glycine conjugate	-0.655 [-1.23 ; -0.08]	0.025	0.484
LysoPC(0:0/20:3)	0.641 [0.07 ; 1.21]	0.028	0.484
S-Adenosylhomocysteine	-0.633 [-1.20 ; -0.06]	0.03	0.484
LysoPC(20:2(11Z,14Z)/0:0)	0.632 [0.06 ; 1.20]	0.03	0.484
Threonic acid	0.631 [0.06 ; 1.20]	0.03	0.484
Malic acid	-0.625 [-1.20 ; -0.05]	0.032	0.484
3-Hydroxycapric acid	-0.609 [-1.18 ; -0.04]	0.037	0.484
2,3,4,5,6,7-Hexahydroxyheptanoic acid	-0.592 [-1.16 ; -0.02]	0.042	0.484
(S)-3-Hydroxyisobutyric acid	-0.579 [-1.15 ; -0.01]	0.047	0.484
LysoPC(0:0/20:5)	0.574 [0.00 ; 1.15]	0.049	0.484
Docosapentaenoic acid (22n-3)	-0.573 [-1.14 ; 0.00]	0.049	0.484

95% CI: 95% confidence interval for regression estimate, *p*-value: raw *p*-value, *p*-corrected: FDR corrected *p*-value

Results are presented only for the metabolites (n = 23) that showed a significant association with CDAI remission at 24 weeks in treatment 2 group. Values are adjusted for age, sex, body mass index (BMI), anti-citrullinated protein antibody (ACPA) status, treatment randomization, current smoking status and DAS28-CRP: Disease activity score using 28 joint counts (CRP-based) at baseline. CDAI: Clinical Disease Activity Index.

Table S7 Results from primary multivariable (adjusted) analysis to assess the association of baseline metabolites with CDAI remission at 24 weeks in treatment 3 group (n = 56)

	Metabolite	Estimate [95% CI]	*p*-value	*p*-corrected
1	gamma-Glutamylmethionine	-0.775 [-1.34 ; -0.21]	0.008	0.719
2	Oleamide	-0.768 [-1.34 ; -0.20]	0.008	0.719
3	gamma-Glutamylisoleucine	-0.754 [-1.32 ; -0.18]	0.009	0.719
4	gamma-Glutamylleucine	-0.743 [-1.31 ; -0.17]	0.01	0.719
5	Hexose	0.668 [0.10 ; 1.24]	0.021	0.719
6	Leucyl-Aspartate / gamma-Glutamylvaline	-0.662 [-1.23 ; -0.09]	0.023	0.719
7	L-Kynurenine	-0.648 [-1.22 ; -0.08]	0.026	0.719
8	5-Hydroxyindoleacetic acid	-0.644 [-1.21 ; -0.08]	0.026	0.719
9	Azelaic acid	-0.642 [-1.21 ; -0.07]	0.027	0.719
10	L-Valine	0.636 [0.07 ; 1.20]	0.028	0.719
11	LysoPC(16:0(OH)/0:0)	-0.636 [-1.20 ; -0.07]	0.028	0.719
12	gamma-Glutamylalanine	-0.605 [-1.17 ; -0.04]	0.037	0.859
13	5-Hydroxy-L-tryptophan	-0.584 [-1.15 ; -0.02]	0.044	0.900
14	S-Adenosylhomocysteine	-0.573 [-1.14 ; 0.004]	0.048	0.900

95% CI: 95% confidence interval for regression estimate, *p*-value: raw *p*-value, *p*-corrected: FDR corrected *p*-value

Results are presented only for the metabolites (n = 14) that showed a significant association with CDAI remission at 24 weeks in treatment 2 group. Values are adjusted for age, sex, body mass index (BMI), anti-citrullinated protein antibody (ACPA) status, treatment randomization, current smoking status and DAS28-CRP: Disease activity score using 28 joint counts (CRP-based) at baseline. CDAI: Clinical Disease Activity Index.

Table S8 Results from primary multivariable (adjusted) analysis to assess the association of baseline metabolites with CDAI remission at 24 weeks in treatment 4 group (n = 54)

**Metabolite**	**Estimate [95% CI]**	***p*-value**	***p*-corrected**
Cortisone	-0.720 [-1.31 ; -0.12]	0.018	0.996
Kynurenic acid	0.672 [0.08 ; 1.27]	0.027	0.996
Norvaline	0.639 [0.04 ; 1.23]	0.035	0.996
Sulfolithocholylglycine	-0.635 [-1.23 ; -0.04]	0.036	0.996
Cytidine	0.617 [0.02 ; 1.21]	0.042	0.996
L-Arginine	0.614 [0.02 ; 1.21]	0.043	0.996
Lidocaine	0.606 [0.01 ; 1.20]	0.046	0.996

95% CI: 95% confidence interval for regression estimate, *p*-value: raw *p*-value, *p*-corrected: FDR corrected *p*-value

Results are presented only for the metabolites (n = 7) that showed a significant association with CDAI remission at 24 weeks in treatment 2 group. Values are adjusted for age, sex, body mass index (BMI), anti-citrullinated protein antibody (ACPA) status, treatment randomization, current smoking status and DAS28-CRP: Disease activity score using 28 joint counts (CRP-based) at baseline. CDAI: Clinical Disease Activity Index.

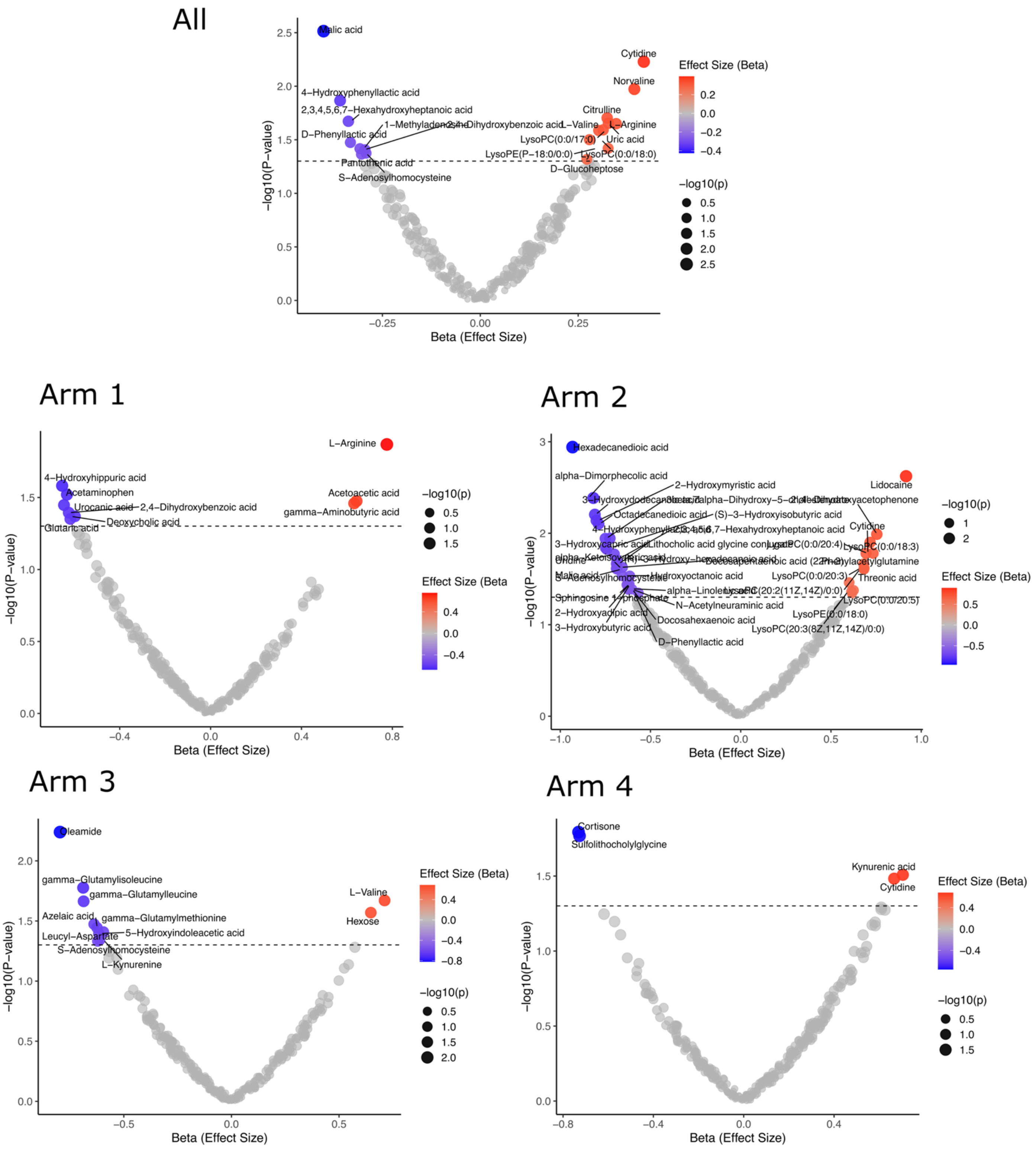

Figure S5 Results from the sensitivity multivariable analysis (multivariable generalized linear model) for association of baseline metabolites with CDAI remission at 24 weeks in overall and four treatment arms (1 to 4). The model is adjusted for age, sex, anti-citrullinated protein antibody status, current smoking status, baseline CDAI (Clinical Disease Activity Index), C-reactive protein, and treatment randomization.

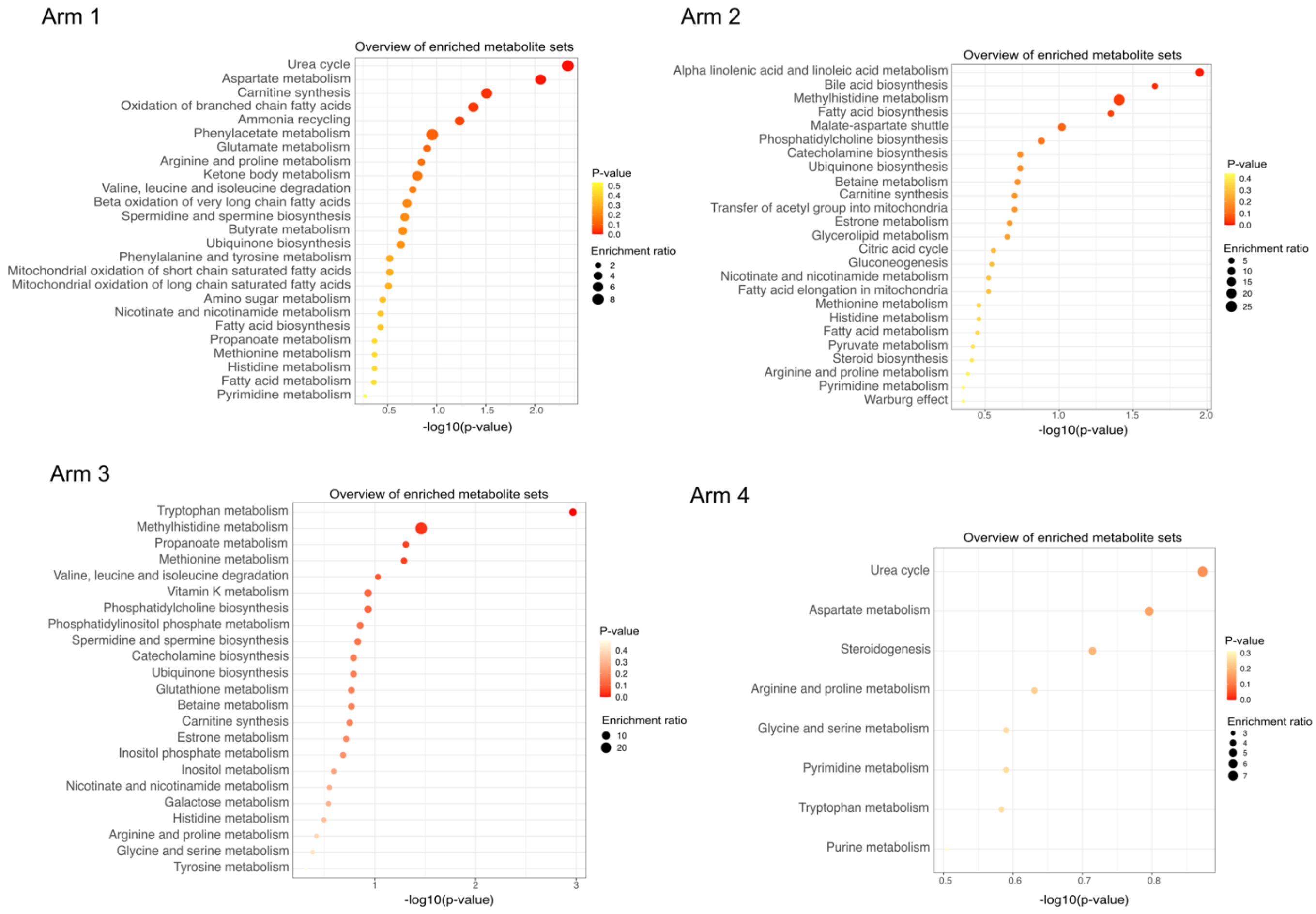

Figure S6 Pathway enrichment analysis plots illustrating significantly perturbed pathways in relation to response to treatment in four treatment arms (1 to 4).

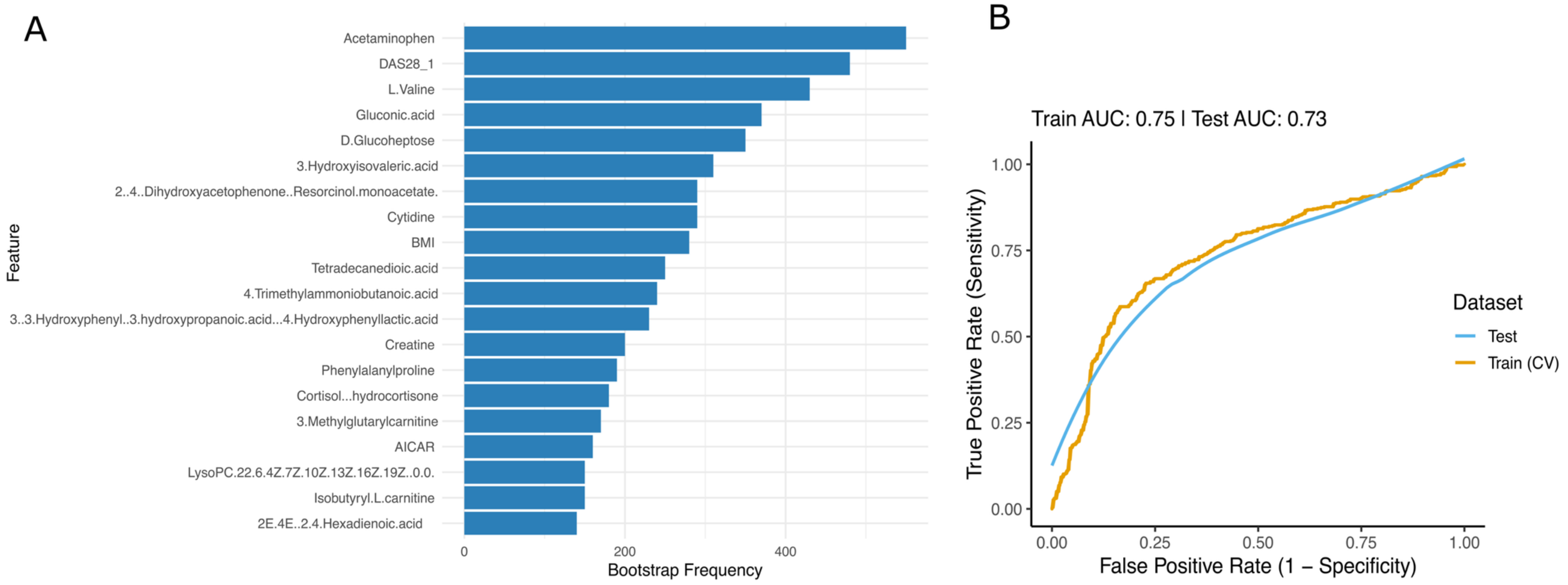

Figure S7 Selection and modeling of potential metabolic and clinical features to discriminate responders from non-responders. A) Frequency of the top 20 features selected across 1,000 bootstraps using the MUVR2 (Multivariate methods with Unbiased Variable selection in R, version 2) algorithm, illustrating robust feature importance, B) Receiver operating characteristic (ROC) curves showing the area under the curve (ROC-AUC) performance of a logistic regression model trained on the top 15 selected features, with results shown for both cross-validated (CV) training and independent test sets.
